# Polarization of M2 macrophages requires Lamtor1 that integrates cytokine and amino-acid signals

**DOI:** 10.1038/ncomms13130

**Published:** 2016-10-12

**Authors:** Tetsuya Kimura, Shigeyuki Nada, Noriko Takegahara, Tatsusada Okuno, Satoshi Nojima, Sujin Kang, Daisuke Ito, Keiko Morimoto, Takashi Hosokawa, Yoshitomo Hayama, Yuichi Mitsui, Natsuki Sakurai, Hana Sarashina-Kida, Masayuki Nishide, Yohei Maeda, Hyota Takamatsu, Daisuke Okuzaki, Masaki Yamada, Masato Okada, Atsushi Kumanogoh

**Affiliations:** 1 Department of Immunopathology, World Premier International Immunology Frontier Research Center, Osaka University, Yamadaoka 2-2, Osaka 565-0871 Japan; 2 Department of Respiratory Medicine, Allergy and Rheumatic Disease, Graduate School of Medicine, Osaka University, Yamadaoka 2-2, Osaka 565-0871, Japan; 3 The Japan Agency for Medical Research and Development—Core Research for Evolutional Science and Technology (AMED-CREST), Gobancho 7, Tokyo 102-0076, Japan; 4 Department of Oncogene Research, Research Institute for Microbial Diseases, Osaka University, Yamadaoka 3-1, Osaka 565-0871, Japan; 5 Department of Neurology, Graduate School of Medicine, Osaka University, Yamadaoka 2-2, Osaka 565-0871, Japan; 6 Department of Pathology, Graduate School of Medicine, Osaka University, Yamadaoka 2-2, Osaka 565-0871, Japan; 7 DNA-chip Development Center for Infectious Diseases, Research Institute for Microbial Diseases, Yamadaoka 3-1, Osaka 565-0871, Japan; 8 Global Application Development Center, Analytical and Measuring Instruments Division, Shimadzu Corporation, Kyoto 604-8511, Japan

## Abstract

Macrophages play crucial roles in host defence and tissue homoeostasis, processes in which both environmental stimuli and intracellularly generated metabolites influence activation of macrophages. Activated macrophages are classified into M1 and M2 macrophages. It remains unclear how intracellular nutrition sufficiency, especially for amino acid, influences on macrophage activation. Here we show that a lysosomal adaptor protein Lamtor1, which forms an amino-acid sensing complex with lysosomal vacuolar-type H^+^-ATPase (v-ATPase), and is the scaffold for amino acid-activated mTORC1 (mechanistic target of rapamycin complex 1), is critically required for M2 polarization. Lamtor1 deficiency, amino-acid starvation, or inhibition of v-ATPase and mTOR result in defective M2 polarization and enhanced M1 polarization. Furthermore, we identified liver X receptor (LXR) as the downstream target of Lamtor1 and mTORC1. Production of 25-hydroxycholesterol is dependent on Lamtor1 and mTORC1. Our findings demonstrate that Lamtor1 plays an essential role in M2 polarization, coupling immunity and metabolism.

## Abstract (web summary)

The role of nutrient-sensing pathways in regulation of innate immune response is unexplored. Here the authors show that IL-4 activates the amino-acid sensing pathway in macrophages and leads to polarization of anti-inflammatory M2 macrophages via the transcription factor liver X receptor.

## Graphical abstract

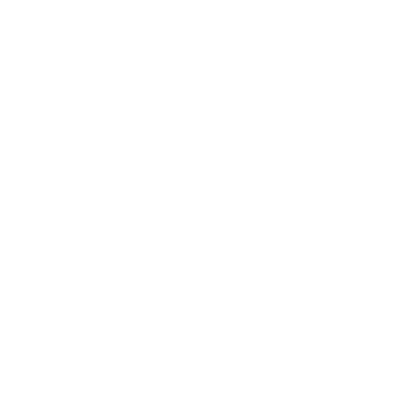

Macrophages are the principal source of innate immune responses that trigger inflammation^1^. In addition, these cells perform homoeostatic functions beyond host defence, including tissue remodelling/repair and orchestration of systemic metabolism^2^. Recently, activated macrophages have been classified as M1 and M2 macrophages^3^. In inflammatory settings, as exemplified by the invasion of pathogenic microorganisms, interferon gamma (IFN-γ) and/or Toll-like receptor ligands activate macrophages as M1 macrophages (M1MΦs), which are implicated in initiating and sustaining inflammation. On the other hand, activation by interleukin-4 (IL-4) or interleukin-13 polarizes macrophages as M2 macrophages (M2MΦs)^3^, which possess anti-inflammatory property and are involved in tissue homoeostasis^4^.

Macrophages also serve as professional phagocytes that engulf dead cells and debris to maintain tissue homoeostasis. In healthy humans, >10 billion cells die each day^5^, and apoptotic cells are engulfed by macrophages. Macrophages digest phagocytosed cells in their abundantly developed lysosomes. Consequently, massive amounts of nutrients, such as amino acids and cholesterol, are continuously produced in the lysosomes of macrophages. The signalling pathway which senses lysosomal amino acids has been revealed recently^6^. Also, cholesterol and its derivatives are sensed to activate the transcription factor liver X receptor (LXR) or to suppress nuclear translocation of sterol regulatory element−binding proteins (SREBPs)^7^. Additionally, it has long been known that engulfment of apoptotic cells suppresses the inflammatory response by macrophages^8^,^9^. Thus, the role of intracellular nutrients and nutrition-sensing pathway in the regulation of macrophages is intriguing.

When intracellular amino acids are present at sufficient levels, the nutrition sensor mechanistic target of rapamycin complex 1 (mTORC1) is recruited from the cytosol to the lysosome membrane to be activated^10^. In addition, it is now clarified that the lysosome membrane is the site at which extrinsic signal and intracellular nutrition sufficiency signal are integrated to induce full activation of mTORC1 (ref. 11). This integration of extrinsic signal and intracellular nutrition sufficiency signal requires the lysosomal adaptor protein complex Ragulator^12^, which consists of Lamtor1, 2, 3, 4 and 5. Among the Lamtor proteins, only Lamtor1 (also known as p18) is attached to the lysosome membrane via covalently bound fatty acids^13^; and the loss of Lamtor1 abolishes amino acid−elicited mTORC1 recruitment to the lysosome^10^.

In this study, we showed that Lamtor1, which forms an amino-acid sensing complex with vacuolar-type H^+^-ATPase (v-ATPase), is essential for the polarization of M2 macrophages and required for the suppression of innate immune response, but dispensable for the polarization of M1 macrophages. IL-4 activated mTORC1 in the presence of Lamtor1 and amino acids, and subsequently polarized macrophages as M2MΦs. Furthermore, we discovered that LXR, a cholesterol−sensing transcription factor, is the downstream target of the amino-acid sensing pathway, including Lamtor1 and mTORC1. Our findings revealed a fundamental coupling between immunity and metabolism, exemplified here by the integration of the extracellular IL-4 signal and the intracellular amino-acid sufficiency signal by Lamtor1, and by the involvement of LXR in the polarization of M2 macrophages.

## Results

### Lamtor1 is essential for polarization of M2 macrophages *in vitro*

Macrophages express the highest amount of Ragulator components (Lamtor1, 2, 3, 4 and 5) and v-ATPase components, compared with other types of cells and tissues (accecible online at BioGPS^14^). We focused on Lamtor1, which anchors the other four Lamtor proteins on lysosome membranes^12^. To investigate the role of Lamtor1 in macrophages, we established conditional knockout (KO) mice lacking Lamtor1 in macrophages (*Lamtor1*^flox/flox^ *LysM-Cre*). To test the system, we generated bone marrow−derived macrophages (BMDMs), and confirmed the complete deletion of Lamtor1 in these cells (Supplementary Fig. 1a–c). In Lamtor1−deficient BMDMs, amounts of other Ragulator components, namely Lamtor2−5, were also reduced (Supplementary Fig. 1c). Subcellular localization of Lamtor1 was the lysosome/late endosome (Supplementary Fig. 1d), as previously reported in HEK293T cells^10^ and mouse fibroblasts^13^. The development of immune system was not affected in 6−8-week-old conditional KO mice. The numbers of monocyte/macrophages, neutrophils, T cells and B cells in the bone marrow and the spleen, and the development of T cells in the thymus, were comparable between conditional KO mice and littermate controls (*Lamtor1*^flox/flox^) (Supplementary Fig. 2).

**Figure 1:**
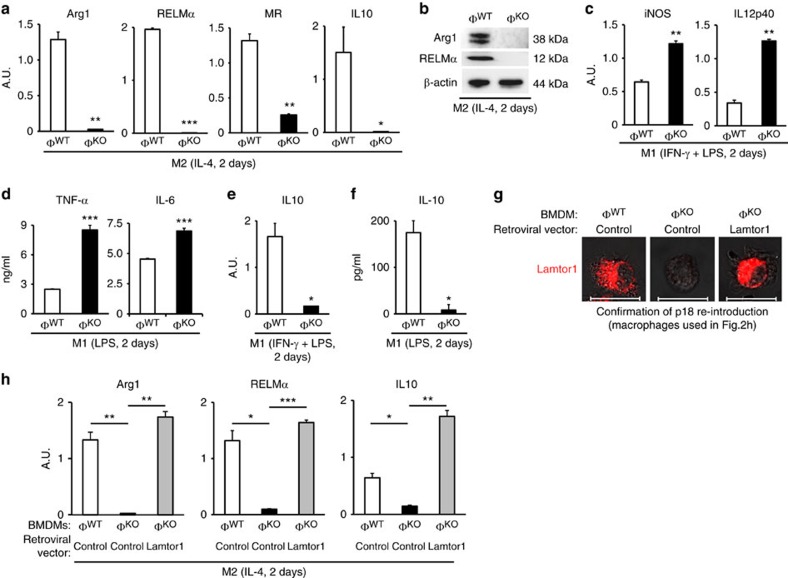
Defective M2 polarization of Lamtor1-deficient macrophages. Expression levels of M2 signature genes in IL-4−stimulated BMDMs were measured by real-time PCR (**a**), and by western blots (**b**). (**c**,**d**) M1 polarization was enhanced in Lamtor1-deficient BMDMs. Expression levels of M1 signature genes were measured by real-time PCR (**c**), and cytokine concentrations in culture supernatants were measured by ELISA (**d**). (**e**,**f**) Expression of the immunosuppressive cytokine IL-10 in M1 (inflammatory) condition was largely reduced in Lamtor1-deficient BMDMs: real-time PCR for the transcript of *IL10* (**e**), and ELISA for IL-10 concentration in culture supernatant (**f**). (**g**) Retroviral transfer of *Lamtor1* gene into Lamtor1-deficient BMDMs restored Lamtor1 protein, confirmed by immunofluorescence. Scale bars indicate 20 μm. See Method section for more detailed information about the gene transfer. (**h**) Real-time PCR showed that re-introduction of Lamtor1 as in (**g**) rescued expression of M2 marker genes, confirming essential role of Lamtor1 in M2 polarization. The control retroviral vector expressed GFP protein. In (**d**,**f**), 100 ng ml^−1^ of LPS was used as stimulant. The representative results of three independent experiments are shown for each panel. Φ^WT^ and Φ^KO^ are defined as in Figure 1. *P<0.05, **P<0.01, ***P<0.001. Error bars show s.d.

Next, we investigated the role of Lamtor1 in polarization of macrophages. BMDMs were differentiated with L929 cell culture supernatant, which contains macrophage colony stimulating factor (M-CSF). BMDMs that lack Lamtor1 showed macrophage characteristics: they were tightly adhesive to culture dish, positive for macrophage surface markers CD11b, F4/80 and MHC class II; and also expressed macrophage marker MerTK (Supplementary Fig. 3). Thus, loss of Lamtor1 did not influence differentiation of macrophages. The Lamtor1-deficient BMDMs, however, exhibited markedly defective polarization to M2MΦs: expression of classic M2 signature genes such as arginase-1 (Arg1), resistin-like molecule alpha (RELMα), mannose receptor (MR) and interleukin-10 (IL10)^3^ was barely detectable neither as mRNA (Fig. 1a) nor protein (Fig. 1b). The defective M2 polarization of Lamtor1-deficient BMDMs after the stimulation by IL-4 was observed both in BMDMs differentiated by L929 cell culture supernatant (Fig. 1a,b) and BMDMs differentiated by recombinant M-CSF (Supplementary Fig. 4). By contrast, polarization as M1MΦs was enhanced in Lamtor1-deficient BMDMs. After stimulation with IFN-γ and lipopolysaccharide (LPS), expression levels of M1 signature genes, inducible nitric oxide synthase (iNOS) and interleukin-12 subunit p40 (IL12p40), were more elevated in Lamtor1-deficient BMDMs than in wild-type counterparts (Fig. 1c). The enhanced M1 polarization was also confirmed the by increased production of pro-inflammatory cytokines from Lamtor1−deficient BMDMs compared with wild-type control BMDMs, following stimulation with LPS (Fig. 1d). Additionally, production of the immunosuppressive cytokine IL-10 during inflammatory response was markedly reduced in Lamtor1−deficient BMDMs: IFN-γ and LPS induced IL-10 expression in control BMDMs, but not in Lamtor1−deficient BMDMs (Fig. 1e). The decreased IL-10 production was also confirmed by enzyme−linked immunosorbent assay (ELISA) (Fig. 1f). Finally, retroviral transfer of the Lamtor1 gene into Lamtor1−deficient BMDMs (Fig. 1g) completely rescued the expression of M2 signature genes, including IL10 (Fig. 1h). Collectively, these data show that Lamtor1 is essential for the polarization of M2MΦs.

### Lamtor1 is required for polarization of M2 macrophages *in vivo*

**Figure 2:**
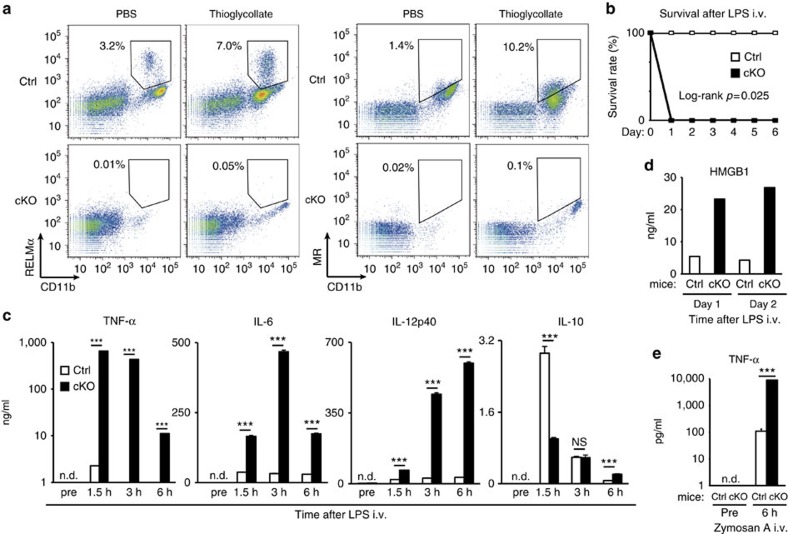
Defective polarization of M2 macrophages and enhanced innate immune response in myeloid−specific Lamtor1 conditional knockout mice. (**a**) M2 macrophages appeared in peritoneal cavity 6 days after the intraperitoneal administration of thioglycollate medium. M2 macrophages (CD11b^high^ RELMα^+^ cells or CD11b^high^ MR^+^ cells; definition of the positive gates are described in Method section) were counted by flow cytometry. Percentage of M2 macrophages in whole peritoneal white blood cells is shown. (**b**–**e**) Enhanced innate immune response in Lamtor1 conditional knockout mice. (**b**) High mortality in Lamtor1 conditional knockout mice after injection of LPS (20 μg per mouse). Three mice were used for each genotype. (**c**) Serum levels of cytokines after intravenous administration of LPS (20 μg per mouse). Time from LPS administration to blood collection is indicated. (**d**) Serum HMGB1 levels at the indicated days after intravenous administration of LPS (5 μg per mouse). (**e**) Serum concentrations of TNF-α after intravenous administration of Zymosan A. Time from Zymosan A administration to blood collection is indicated. Concentrations of each cytokine and HMGB1 were measured by ELISA. Ctrl: littermate *Lamtor1*^flox/flox^; cKO: *Lamtor1*^flox/flox^ *LysM-Cre* mice. Samples were measured in triplicate (**c**,**e**). The representative results of three independent experiments are shown (**a**,**c**). ***P<0.001. Error bars show s.d.

We next investigated whether Lamtor1 was required for *in vivo* polarization of M2MΦs. In a mouse model of aseptic peritonitis, M2MΦs expressing M2 markers RELMα or MR accumulated in peritoneal cavities of control mice 6 days after the intraperitoneal injection of thioglycollate medium (Fig. 2a). In this peritonitis model, mRNAs obtained from whole peritoneal white blood cells showed upregulation of M2 markers Arg1 and MR, and little upregulation of M1 markers iNOS and TNF-α (Supplementary Fig. 5). Indeed, relative expression level of Arg1 to a control house keeper gene (GAPDH) in these peritoneal-elucidated cells at healing stage of peritonitis was as high as that in *in vitro*-activated M2MΦs by recombinant IL-4 (Supplementary Fig. 5). The upregulation of MR, RELMα and Arg1 supported that M2 polarization is elicited on day 6 in this sterile peritonitis model. Our model, in which sterile inflammation induced the polarization of M2MΦs, is also compatible with a recent report that showed the induction of peritoneal M2MΦs by sterile liver damage^15^. In our aseptic peritonitis model, M2MΦs was not induced in Lamtor1 conditional KO mice in contrast to littermate control mice (Fig. 2a).

Next, to examine the importance of Lamtor1 in innate immune response *in vivo*, we used an LPS-induced sepsis model. Although the administered dose was non-lethal for their genetic background C57BL/6 mice, none of the Lamtor1 conditional KO mice survived for 1 day after the intravenous administration of LPS (Fig. 2b). Indeed, LPS injection resulted in markedly severe hypercytokinemia in Lamtor1 conditional KO mice: the maximum serum level of TNF-α was >100−fold higher, and the maximum levels of IL-6 and IL-12p40 were >10−fold higher than that were observed in littermate control mice (Fig. 2c). By contrast, the maximum serum level of IL-10 in the conditional KO mice was lower than that in control mice (Fig. 2c), suggesting that Lamtor1 also plays an important role in the prevention of excessive inflammatory response. High-mobility group box 1 protein (HMGB1), a late mediator of septic death^16^, was also produced at higher levels in Lamtor1 conditional KO mice than in littermate control mice (Fig. 2d). This hyper-responsiveness was also observed when conditional KO mice were challenged with a TLR2 ligand Zymosan A (Fig. 2e). Overall, myeloid-specific Lamtor1 conditional KO mice exhibited defective polarization of *in vivo* M2MΦs, reduced IL-10 production, and an enhanced innate immune response.

### Lamtor1 and mTORC1 are required for M2 polarization

**Figure 3:**
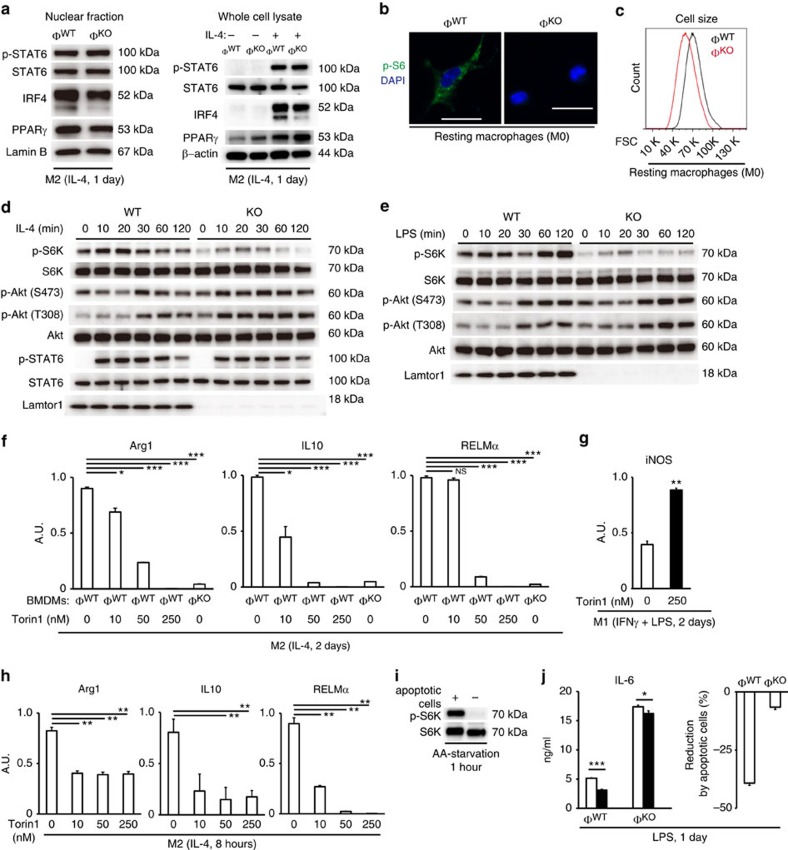
Lamtor1 is required for IL-4−elicited activation of mTORC1, which is necessary for M2 polarization. (**a**) Western blots for previously known essential M2−polarizing factors in IL-4−stimulated BMDMs. See Supplementary Fig. 8a for confirmation of the fractionation. (**b**) Immunofluorescence confocal microscopy for phospho-S6 ribosomal protein indicated decreased mTORC1 activity in Lamtor1-deficient BMDM. Scale bars indicate 20 μm. (**c**) Flow cytometry showed that Lamtor1-deficient BMDMs were smaller in cell sizes than wild-type BMDMs. (**d**) Time course for acute activation of mTORC1, mTORC2 and JAK-STAT6 pathway during the stimulation by IL-4 (that is, M2-polarizing condition). (**e**) Time course for acute activation of mTORC1 and mTORC2 in BMDMs stimulated by 100 ng ml^−1^ of LPS. (**f**–**h**) Inhibition of mTORC1 by an mTOR inhibitor selectively abolished M2 polarization, but did not block M1 polarization. (**f**) Real-time PCR for M2 signature genes. M2 macrophages were polarized with IL-4 stimulation and concomitant mTOR inhibition. See Supplementary Fig. 8b for exclusion of unintended suppressive effect of the vehicle. (**g**) Real-time PCR for an M1 signature gene in wild-type BMDMs. M1 macrophages were polarized with IFNγ and LPS stimulation and concomitant mTOR inhibition. (**h**) Real-time PCR showed that M2 polarization of wild-type BMDMs was blocked even at 8 h of IL-4 stimulation and concomitant inhibition of mTOR. (**i**,**j**) Engulfed apoptotic cells activated mTORC1, and decreased IL-6 production from LPS-stimulated BMDMs. (**i**) BMDMs that had phagocytosed apoptotic thymocytes showed active mTORC1, even in the absence of amino acids in culture medium. Western blots for phosphorylation of S6K are shown as mTORC1 activity. (**j**) BMDMs that had phagocytosed apoptotic thymocytes showed decreased production of IL-6 after stimulation by LPS (100 ng ml^−1^) in amino acids-free medium. IL-6 concentration measured by ELISA (left panel), and calculated percentage of the decrease (right panel) are shown. In left panel, BMDMs that did not engulf apoptotic thymocytes are shown as white bars, and ones that engulfed apoptotic thymocytes are shown as black bars. Φ^WT^ and Φ^KO^ are defined as in Fig. 1. The representative results of three independent experiments are shown (**a**–**g**). *P<0.05, **P<0.01, ***P<0.001. Error bars show s.d.

Phosphorylation of STAT6 and induction of IRF4 by IL-4 are required for polarization of M2MΦs (ref. 3). The nuclear levels of phosphorylated STAT6 and IRF4, however, were comparable between Lamtor1-deficient and wild-type BMDMs (Fig. 3a). Nuclear localization of PPARγ, which is required for expression of Arg1 and MR (ref. 17), was also comparable (Fig. 3a). These results prompted us to search another downstream signalling pathway that is critically required for M2 polarization as similar to Lamtor1.

**Figure 4:**
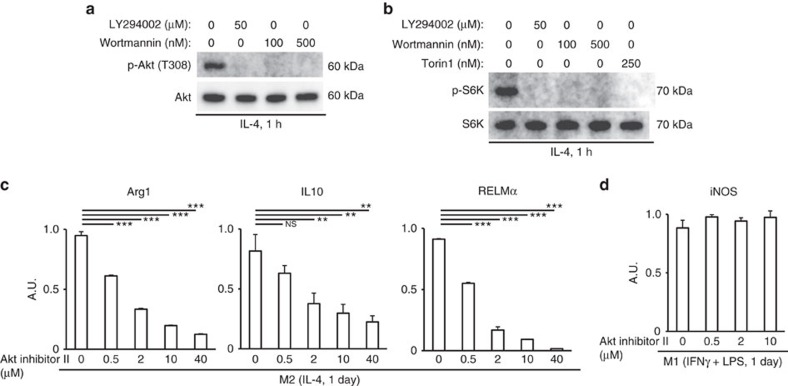
PI3K and Akt are the upstream signal pathway responsible for mTORC1 activation by IL-4. (**a**) Western blot for Akt phosphorylation in wild-type BMDMs stimulated for 1 h with IL-4. Inhibitors for PI3K were added 10 min before IL-4 stimulation, and also existed during IL-4 stimulation. (**b**) Western blot for mTORC1 activity in wild-type BMDMs. Macrophages were stimulated for 1 h with IL-4. Each inhibitor for PI3K or mTOR was added 10 min before IL-4 stimulation, and also existed during IL-4 stimulation. (**c**,**d**) Real-time PCR for M2 (**c**) and M1 (**d**) signature genes expressed in wild-type BMDMs polarized with each stimulants with concomitant Akt inhibition. Polarizing conditions in detail are described in Method section. The representative results of two independent experiments are shown for each panel. **P<0.01, ***P<0.001. Error bars show s.d.

Because Lamtor1 is the scaffold for mTORC1 activation^10^, we measured mTORC1 activity in macrophages. Activity of mTORC1, measured by phosphorylation of S6 ribosomal protein (p-S6), was reduced in Lamtor1-deficient BMDMs at resting state (‘M0' macrophage) (Fig. 3b). The reduced mTORC1 activity in resting Lamtor1-deficient BMDMs was also supported by their smaller cell sizes than that of wild-type BMDMs (Fig. 3c), as reported in previous studies that showed lower mTORC1 activity decreased cell size^12^,^18^. Next, we measured mTORC1 activity in M2-polarizing condition. During activation by IL-4, phosphorylation level of p70 S6 kinase (S6K) was lower in Lamtor1-deficient BMDMs than that in wild-type BMDMs (Fig. 3d). In the same activating condition, phosphorylation of Akt (Serine 473) by mTORC2 was slightly increased in Lamtor1-deficient BMDMs (Fig. 3d); it was consistent with the reduced mTORC1 activity in them. In contrast, early activation of JAK-STAT6 pathway by IL-4 was not affected, shown by the comparable phosphorylation levels of STAT6 between wild-type and Lamtor1-deficient BMDMs (Fig. 4d). Also in M1-polarizing condition, mTORC1 activity in LPS-stimulated Lamtor1-deficient BMDMs was lower than that in LPS-stimulated wild-type BMDMs (Fig. 3e). Taken altogether, these data indicated that mTORC1 activity in Lamtor1-deficient BMDMs was lower than that in wild-type BMDMs, both before (M0MΦs) and during M1- or M2-polarizing activations.

Accordingly, we hypothesized that Lamtor1-mediated activation of mTORC1 is required for M2 polarization. Although the allosteric mTORC1 inhibitor rapamycin did not block the polarization of M2 macrophages (Supplementary Fig. 6), the ATP-competitive mTOR inhibitor Torin1 (ref. 19) blocked the expression of M2 signature genes Arg1, IL10 and RELMα in dose-dependent manner (Fig. 3f). By contrast, expression of the M1 signature gene iNOS was rather enhanced by Torin1 treatment (Fig. 3g). This selective requirement of mTOR activity for M2 polarization was reminiscent of the selective requirement of Lamtor1 for M2 polarization. To exclude off-target effects resulting from long-time inhibition of mTOR, we performed IL-4 stimulation and concomitant mTOR-inhibition for as short as 8 h. M2 polarization was again diminished with this experimental condition (Fig. 3h).

In addition, we found that phagocytosed apoptotic cells activate mTORC1 in BMDMs (Fig. 3i), even they were cultured in amino-acid starvation media. Also, wild-type BMDMs that had phagocytosed apoptotic cells showed decreased production of IL-6 after LPS stimulation (Fig. 3j). These data suggested the possible roles of apoptotic cells to activate mTORC1, and to prevent excessive inflammatory response.

In regard to the upstream signal pathway for mTORC1, it is reported that mTORC1 activation by IL-4 is mediated by the phosphoinositide 3-kinase (PI3K)–Akt pathway^20^,^21^. In concordance with previous reports, two PI3K inhibitors, Wortmannin and LY294002, inhibited phosphorylation of both Akt (Threonine 308) and S6K (Fig. 4a,b), confirming the involvement of the PI3K−Akt pathway in mTORC1 activation by IL-4. Inhibition of Akt by Akt inhibitor II abolished the expression of M2 signature genes, but did not affect expression of an M1 signature gene (Fig. 4c,d). These data indicated that full activation of mTORC1 by IL-4 cannot be elicited in Lamtor1-deficient macrophages, resulting in defective M2 polarization of them.

### Lysosomal amino acid-sensing complex in M2 polarization

**Figure 5:**
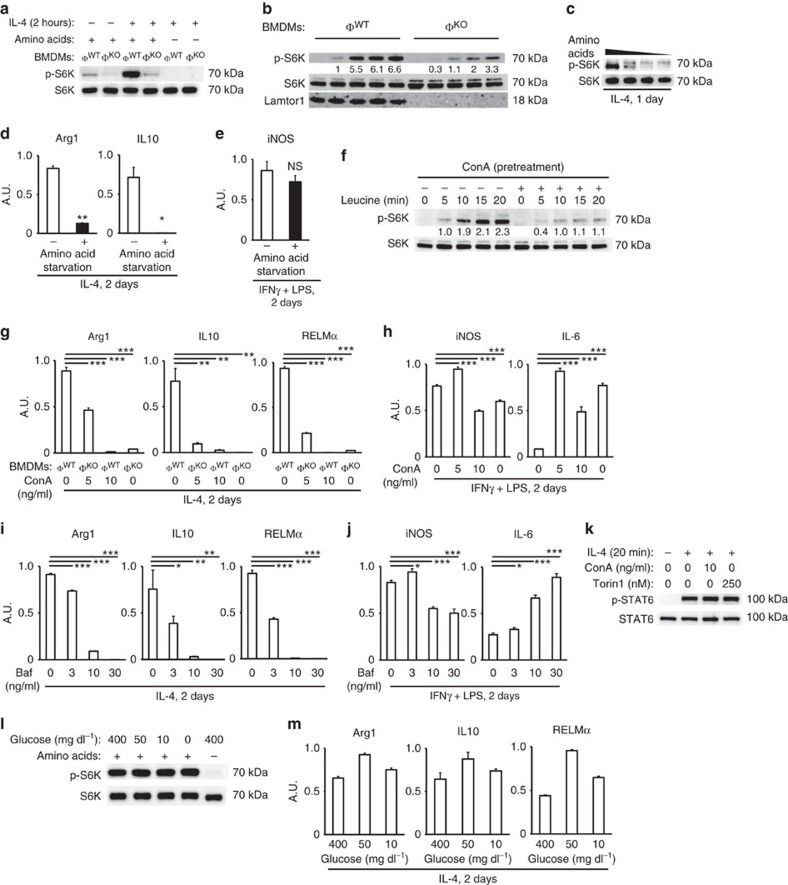
Amino-acid sensing machineries are required for M2 polarization. (**a**) Western blot for phosphorylated S6 kinase showed that full activation of mTORC1 by IL-4 requires both Lamtor1 and amino acids. (**b**) Western blots for leucine−elicited mTORC1 activation. After starved 1 h for amino acids, BMDMs were stimulated with leucine (2 mg ml^−1^). (**c**) Western blots for phospho-S6 kinase in wild-type BMDMs stimulated with IL-4 in amino-acid starvation media. (**d**,**e**) Macrophage polarization in amino acid starvation media. Real-time PCR for M2 (**d**) and M1 (**e**) signature genes in amino acid−starved wild-type BMDMs are shown. (**f**–**j**) Involvement of v-ATPase in amino-acid sensing and M2 polarization. (**f**) Western blots for leucine-elicited mTORC1 activation. After starved 1 h for amino acids, BMDMs were stimulated with leucine (2 mg/ml). Concanamycin A (ConA) was added 10 min before leucine, and washed out just before the addition of leucine to media. (**g**–**j**) Real-time PCR for M2 (**g**,**i**) and M1 (**h**,**j**) signature genes in wild-type BMDMs polarized with each stimulant and concomitant ConA or Bafilomycin A1 (Baf). See Supplementary Fig. 8c for exclusion of unintended suppressive effect of the vehicle. (**k**) Western blots for STAT6 phosphorylation in wild-type BMDMs treated with IL-4 and concomitantly used inhibitors for v-ATPase or mTOR. (**l**) Western blots for mTORC1 activity. Amino acid−starvation (1 h) abolished phosphorylation of S6 kinase, but glucose starvation (1 h) did not. (**m**) Real-time PCR for M2 marker genes expressed in wild-type BMDMs cultured in low-glucose media. Φ^WT^ and Φ^KO^ are defined as in Fig. 1. The representative results of three independent experiments are shown for each panel. *P<0.05, **P<0.01, ***P<0.001. Error bars show s.d.

Because Lamtor1 is involved in amino acid−elicited activation of mTORC1 (ref. 10), we next investigated the role of amino-acid sensing machinery in polarization of M2MΦs. Full activation of mTORC1 by IL-4 was dampened either by the absence of Lamtor1 or amino acids (Fig. 5a). In regard to amino acid-sensing, activation of mTORC1 by leucine^22^ was impaired in Lamtor1-deficient macrophages (Fig. 5b), confirming the involvement of Lamtor1 in amino-acid sensing. We further confirmed that mTORC1 activity in wild-type BMDMs was abolished after 1 day of IL-4 stimulation and concomitant amino-acid starvation (Fig. 5c). To test the role of amino acid−elicited signals in M2 polarization, we polarized BMDMs in amino-acid starvation media. Polarization of M2MΦs, but not M1MΦs, was abolished by amino-acid starvation (Fig. 5d,e).

Next, we investigated the involvement of v-ATPase in M2 polarization. Recent work showed that v-ATPase co-localizes with Lamtor1 and is required for amino acid−elicited activation of mTORC1 at lysosome^18^. Pre-teatment of macrophages with the v-ATPase inhibitor concanamycin A decreased activation of mTORC1 by leucine (Fig. 5f), confirming the involvement of v-ATPase in amino-acid sensing. Moreover, the polarization of M2MΦs was diminished by concanamycin A in a dose-dependent manner (Fig. 5g). On the other hand, polarization of M1MΦs was not abolished by concanamycin A, even at the concentration that completely blocked M2 polarization (Fig. 5h). Similarly, another v-ATPase inhibitor bafilomycin A1 blocked expression of M2 signature genes Arg1, IL10 and RELMα, but did not abolish expression of M1 signature genes iNOS and IL6 (Fig. 5i,j). The unimpaired phosphorylation of STAT6 in v-ATPase−inhibited or mTOR−inhibited wild-type BMDMs that were stimulated with IL-4 (Fig. 5k) again supported that this amino-acid sensing pathway constitutes a separate signalling axis from previously known JAK−STAT6 pathway. In contrast to amino acids, glucose was dispensable for IL-4−elicited mTORC1 activation (Fig. 5l). Indeed, glucose starvation did not show any apparent effect on M2 polarization (Fig. 5m).

To summarize, we showed the dual roles of Lamtor1 and v-ATPase in both amino acid-sensing and IL-4−elicied macrophage activation. Thus, Lamtor1 is the integrator of amino-acid sufficiency signal and IL-4 signal, and is required for full activation of mTORC1 and subsequent polarization of M2MΦs.

### Gene expression by LXR requires Lamtor1 and mTOR

**Figure 6:**
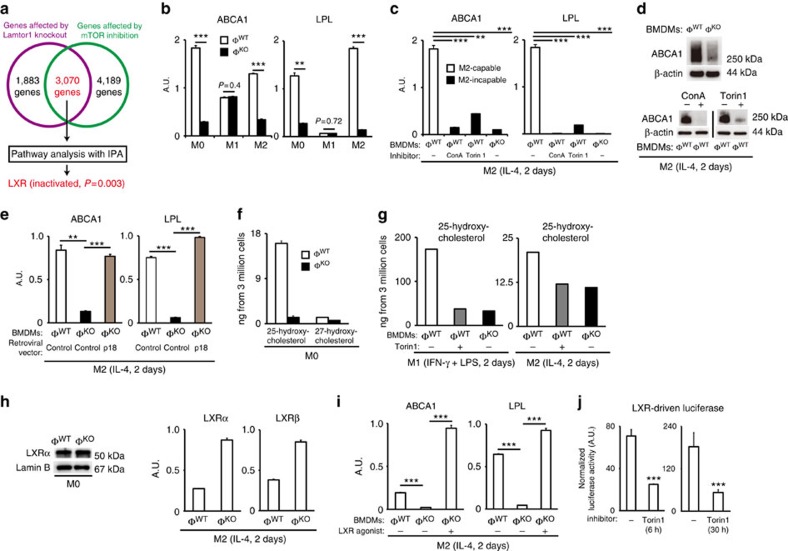
LXR is the downstream target of Lamtor1 and mTOR. (**a**) Strategy to find the downstream pathway of Lamtor1 and mTORC1. Numbers of genes whose expression levels were changed equal or more than 2-fold in gene expression microarray are shown as a Venn diagram. The 3,070 genes affected by both Lamtor1-loss and mTOR-inhibition were input to gene enrichment analysis program in a commercially available pathway analysis software IPA; Inactivation of LXR was suggested. See Supplementary Data 1 for the list of genes in each group. (**b**) Real-time PCR for LXR-target genes ABCA1 and LPL. M0 BMDMs were at resting condition; M1 BMDMs were stimulated with 20 ng ml^−1^ of IFN-γ and 100 ng ml^−1^ of LPS for 2 days; M2 BMDMs were stimulated with 50 ng ml^−1^ of IL-4 for 2 days. (**c**) Real-time PCR for LXR−target genes. Expression of these genes was decreased in BMDMs that could not polarize to M2MΦs. (**d**) Western blot for ABCA1. Knockout of Lamtor1, treatment with a v-ATPase inhibitor or an mTOR inhibitor decreased expression of ABCA1 protein in IL-4−stimulated BMDMs. (**e**) Real-time PCR for LXR-target genes. Re-introduction of Lamtor1 gene into Lamtor1-deficient BMDMs rescued expression of ABCA1 and LPL during M2-polarizing stimulation. The control retroviral vector expressed GFP protein. (**f**) Intracellular amounts of endogenous LXR ligands oxysterols were measured by mass spectrometry. (**g**) Intracellular amounts of 25-hydroxycholesterol were measured after M1- of M2-polarizing stimulation. (**h**) Western blot for nuclear LXRα in M0 macrophages (left panel); and real-time PCR for LXRα and LXRβ in IL-4−stimulated BMDMs. (**i**) Real-time PCR showed that an LXR agonist GW3965, used in combination with its obligatory heterodimer RXR's ligand 9-cis retinoic acid, rescued expression of LXR-target genes in Lamtor1-deficient BMDMs. (**j**) Luciferase assay in HEK293 cells showed that mTOR is required for LXR activity. Cells transfected with LXR−driven luciferase and control constitutively-active luciferase were treated with an mTOR inhibitor. ConA: concanamycin A. Φ^WT^ and Φ^KO^ are defined as in Fig. 1. *P<0.05, **P<0.01, ***P<0.001. Error bars show s.d.

To further clarify how Lamtor1 and mTORC1 promote M2 polarization of macrophages, we searched for the downstream nuclear factor of Lamtor1 and mTORC1. Gene expression microarray was performed with RNA that had been obtained from BMDMs stimulated for 2 days. Wild-type BMDMs that was stimulated with IL-4, Lamtor1-deficient BMDMs stimulated with IL-4, and wild-type BMDMs stimulated with both IL-4 and concomitantly used Torin1 (250 nM) were used as samples. Genes whose expression levels were changed equal or >2-fold both by Lamtor1-loss and Torin1-treatment were input to gene enrichment analysis, using a commercially available software Ingenuity Pathway Analysis. As a result, decreased expression of LXR-target genes was highlighted in the three most significantly affected pathways (schematically depicted in Fig. 6a).

Real-time PCR confirmed that expression of LXR-target genes, ABCA1 and LPL (ref. 7), were decreased in Lamtor1-deficient BMDMs: expression levels were markedly different between wild-type and Lamtor1-deficient BMDMs in M0 and M2 conditions, but were comparable in M1 condition (Fig. 6b). Consistently, expression levels of these LXR-target genes in IL-4−stimulated BMDMs were reduced also in mTOR−inhibited wild-type BMDMs and v-ATPase−inhibited wild-type BMDMs (Fig. 6c). Decreased expression of ABCA1 protein in these macrophages was also confirmed by western blot (Fig. 6d). These results suggested that LXR is the downstream transcription factor of Lamtor1 and mTORC1 in macrophages. Moreover, transcription of ABCA1 and LPL was rescued by re-introduction of Lamtor1 gene into Lamtor1-deficient BMDMs (Fig. 6e). These data suggested that gene expression by LXR requires Lamtor1, v-ATPase and mTORC1.

**Figure 7:**
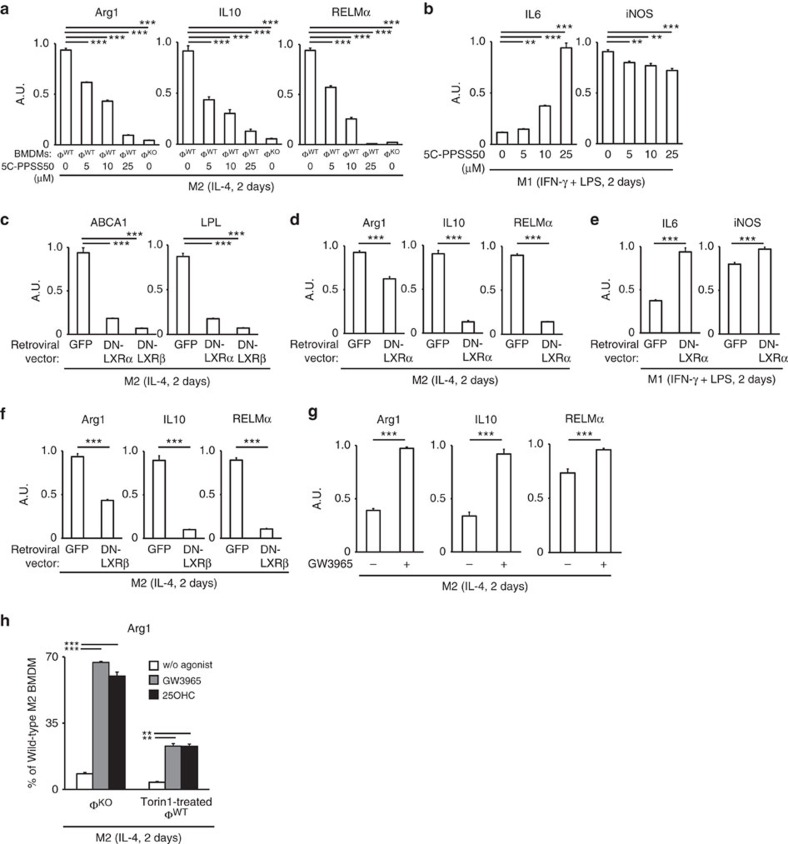
Active LXR is required for M2 polarization. (**a**,**b**) Real-time PCR for M2 (**a**) and M1 (**b**) signature genes in BMDMs polarized with each stimulants and concomitantly used LXR antagonist. See Supplementary Fig. 8d for exclusion of unintended suppressive effect of the vehicle. (**c**–**f**) Overexpression of dominant-negative (DN) LXRα and LXRβ in wild-type BMDMs and its effect on gene expression. (**c**) Real-time PCR for LXR-target genes confirmed the suppressive activity of DN-LXRα and DN-LXRβ. (**d**–**f**) Real-time PCR for M2- (**d**, **f**) and M1- (**e**) marker genes. (**g**) Real-time PCR showed that concomitant stimulation with synthetic LXR agonist GW3965 and IL-4 increased expression of M2 marker genes in wild-type BMDMs, compared with IL-4 stimulation. (**h**) GW3965 or an LXR ligand 25-hydroxysterol rescued M2 polarization of both Lamtor1-deficient BMDMs and mTOR-inhibited wild-type BMDMs. Φ^WT^ and Φ^KO^ are defined as in Fig. 1. The representative results of three independent experiments are shown for each experimental panel. *P<0.05, **P<0.01, ***P<0.001. Error bars show s.d.

LXR is a ligand-dependent transcription factor, whose endogenous ligands are oxysterols^7^. To further elucidate the mechanism how Lamtor1 promotes expression of LXR-target genes, we measured cellular amounts of oxysterols in BMDMs. Among oxysterols, namely 22(R)-hydroxycholesterol, 24(S)-hydroxycholesterol, 25-hydroxycholesterol and 27-hydroxycholesterol, the most abundant oxysterol in wild-type BMDMs was 25-hydroxycholesterol (Fig. 6f). Small amount of 27-hydroxycholesterol, and neither 22(R)-hydroxycholesterol nor 24(S)-hydroxycholesterol, was detected by mass spectrometry (Fig. 6f). The amount of 25-hydroxycholesterol in Lamtor1-deficient BMDMs was much smaller than that in wild-type BMDMs before macrophage activation (Fig. 6f). Additionally, Lamtor1-deficient BMDMs and Torin1-treated wild-type BMDMs after 2 days of M1 or M2-polarizing stimulations contained lower amounts of 25-hydroxycholesterol than wild-type counterparts did (Fig. 6g). We also found that intracellular amount of 25-hydroxysterol in M1-state wild-type BMDMs was about 10 times larger than that in M0 or M2-state wide-type BMDMs (Fig. 6f,g). By contrast, the amounts of LXR protein in M0-state wild-type and Lamtor1-deficient BMDMs were comparable (Fig. 7h), and mRNA levels of LXRs in M2-state Lamtor1-deficient BMDMs were not less than that in wild-type counterparts (Fig. 6h). These data suggested that Lamtor1 and mTORC1 is required for production of 25-hydroxycholesterol, and subsequent activation of LXR in macrophages. To confirm this molecular link between Lamtor1 and LXR more robustly, we treated BMDMs in M2-polarizing condition with a synthetic LXR agonist GW3965 and 9-cis retinoic acid (a ligand for LXR's obligatory partner RXR^7^). The treatment completely rescued expression of LXR-target genes in Lamtor1-deficient BMDMs (Fig.6i). These data indicated that Lamtor1 and mTOR is required for production of 25-hydroxycholesterol, and that the dependence of LXR activity on Lamtor1 and mTOR becomes apparent in M0 and M2 state macrophages presumably by the relatively small amounts of 25-hydroxychoesterol in these macrophages.

Additionally, in HEK293 cells, LXR-driven expression of firefly luciferase, normalized against constitutively expressed renilla luciferase, was reduced following both short and long treatment with Torin1, again indicating that mTOR is required for transcription by LXR (Fig. 6j). The result suggests that the dependence of LXR transcriptional activity on mTOR may be applicable not only to macrophages, but also to some other types of cells.

### Active LXR is required for polarization of M2MΦs

We focused on LXR as the candidate transcription factor that links Lamtor1 and M2 polarization of macrophages, because Lamtor1-loss, mTOR-inhibition and v-ATPase inhibition had uniformly suppressed gene expressions of both classic LXR-target genes and M2 signature genes (Figs 1a and 3f, Figs 5i and 6c). The essential requirement of Lamtor1 for expressions of both M2 marker genes (Fig. 1h) and LXR-target genes (Fig. 6e) further supported this hypothesis. Because the expressions of LXR-target genes and M2 marker genes were controlled in a parallel manner depending on Lamtor1 and active mTORC1, it prompted us to examine whether LXR activity and M2 marker expression have a causal relationship. To test the working hypothesis that active LXR is the necessary factor for M2 polarization, we treated wild-type BMDMs with an LXR antagonist 5C-PPSS50 (ref. 23) during M2 polarization by IL-4. The antagonist abolished the expression of the M2 signature genes Arg1, IL10 and RELMα, but did not abolish the expression of the M1 signature genes iNOS and IL6 (Fig. 7a,b). This selective and complete blocking of M2 polarization with the LXR antagonist was reminiscent of the selective impairment of M2 polarization observed in Lamtor1-deficient or mTOR-inhibited BMDMs. Moreover, dominant-negative LXRα (ref. 24) blocked M2 polarization of wild-type macrophages, but did not block M1 polarization (Fig. 7c–e). Dominant-negative LXRβ (ref. 24) also blocked M2 polarization of wild-type BMDMs (Fig. 7c,f). Additionally, an LXR agonist GW3965 increased expression of M2 marker genes (Fig. 7g) in wild-type BMDMs stimulated with IL-4. Thus, the loss of LXR activity led to the defective M2 polarization, and the pharmacological activation of LXR led to the increased expression of M2 marker genes; it suggests that expression levels of M2 marker genes are determined by LXR activity. Moreover, an endogenous LXR ligand 25-hydroxycholesterol or synthetic LXR agonist GW3965 partially rescued M2 polarization of mTOR-inhibited wild-type BMDMs and Lamtor1-deficient BMDMs: expression level of arginase-1 in Lamtor1-deficient BMDMs was rescued up to 70% of that in wild-type M2MΦs by these LXR ligands (Fig. 7h). Expression levels of another M2 marker IL10 in Torin1-treated wild-type BMDMs and Lamtor1-deficient BMDMs were rescued up to around 30% of the wild-type counterparts (Supplementary Fig. 7). The incomplete rescue with these LXR ligands implicated the presence of other important factors that are affected by the loss of Lamtor1 or mTOR activity. Thus, M2 polarization requires active LXR as a necessary factor, but LXR is not sufficient for M2 polarization. Collectively, these findings indicated that LXR, which is activated downstream of Lamtor1 and mTOR via 25-hydroxycholesterol, is necessary for polarization of M2MΦs.

## Discussion

In this study, we revealed dual roles of Lamtor1, v-ATPase and mTORC1 in both amino acid sensing and M2 polarization. In regard to the role of metabolism in macrophage activation, previous studies showed that glycolysis is important for M1MΦs^25^, and fatty-acid oxidation is important for M2MΦs^25^. To our knowledge, the importance of amino acids or amino acid−elicited mTORC1 activation in macrophage polarization has not been studied^26^. A very recent paper^27^ reported decreased expression of some M2 marker genes in amino-acid starved and IL-4-stimulated macrophages, a compatible observation with our results. Our results showed that Lamtor1 was required not only for amino-acid sensing, but also for full activation of mTORC1 by both amino acids and IL-4. We also found that Lamtor1 is critically required for M2 polarization, but dispensable for M1 polarization. Next, we have expanded the critical role of Lamtor1 in M2 polarization of macrophages to its adjacent protein complex v-ATPase, and their downstream signalling kinase-complex mTORC1. Inhibition of v-ATPase, inhibition of mTORC1, and amino-acid starvation reproduced the selective blocking of M2 polarization, as found in Lamtor1-deficient macrophages. Thus, we revealed the critical roles of amino acids and its sensing machinery in M2 polarization of macrophages. In addition to the investigation on intracellular molecular mechanism of Lamtor1 in M2 polarization, we showed the role of Lamtor1 in polarization of M2MΦs at the healing stage of aseptic peritonitis model, and also showed that loss of Lamtor1 in macrophages led to the marked hypercytokinemia and high mortality in a mouse sepsis model. Our present study, thus, newly adds the Lamtor1-containing amino-acid sensing signal pathway to one of the critical signal pathways involved in activation of macrophages.

In regard to the role of the intracellular nutrition sensor mTORC1 in macrophage activation, our conclusions are compatible with the results of a previous study^28^, which showed that human monocytes treated with an mTOR inhibitor rapamycin produced 2-fold more pro-inflammatory cytokines and 2-fold less IL-10. By contrast, in another study, BMDMs in which mTORC1 was constitutively active due to the loss of the suppressor TSC1 exhibited reduced expression of M2 signature genes^20^. Those authors attributed the weakened M2 polarization in TSC1-deficient macrophages to suppression of Akt by constitutively and excessively activated mTORC1. In our data, however, acute suppression of mTORC1 completely blocked M2 polarization. We suppose two possible explanations for this discrepancy. One is that the strength of mTORC1 activity should be fine-tuned during M2 polarization. That is, M2 polarization may require mTORC1 activation in physiologically appropriate range, thus, constitutive and excessive activation of mTORC1 by the loss of TSC1 altered intracellular milieu to be resistant to M2 polarization. Another possible explanation is that mTORC1 plays a yet undiscovered and Lamtor1-dependent effector role at the lysosome membrane during M2 polarization. Our data that the activity of mTORC1 was diminished but still present in Lamtor1-deficient macrophages favour this possibility. Although the known role of Lamtor1 for now is the scaffold for mTORC1 activation in amino acid-sufficient condition, the possibility that mTORC1, which is recruited to lysosome under the presence of amino acids, acts as an effector with Lamtor1 at the lysosome membrane would be an intriguing next research. In addition, the proliferative nature of M2MΦs^29^ supports our conclusion that mTORC1 is required for M2 polarization, because cell proliferation requires accelerated production of proteins, lipids and nucleic acids, all which requires active mTORC1 (ref. 30). Thus, we conclude that activation of mTORC1, which should be in an appropriate range and duration, is required for M2 polarization of macrophages.

As a novel signalling pathway, we found that the transcription factor LXR is a downstream target of Lamtor1 and mTORC1 in macrophages. First, we showed that Lamtor1, v-ATPase and mTORC1 are required for the gene expression by LXR, using established LXR-target genes ABCA1 and LPL as read-out. We next identified the molecular mechanism how Lamtor1 and mTORC1 control LXR activity: production of endogenous LXR ligand 25-hydroxycholesterol was dependent on Lamtor1 and active mTORC1. We further identified LXR as the transcription factor that links Lamtor1-mTORC1 and expression of M2 marker genes Arg1, IL10 and RELMα. Involvement of active LXR in M2 polarization was proven not only by the fact that expression levels of LXR-target genes and M2 marker genes were changed in a parallel manner, but also by the facts that inactive LXR blocked expression of M2 marker genes and that pharmacological activation of LXR increased expression of M2 marker genes. In compatible with our results, previous reports^31^,^32^ showed increased expressions of M2 marker genes Arg1, MR and RELMα in the macrophages treated with LXR agonists. There is, however, an apparently conflicting report that pretreatment of BMDM with an LXR agonist GW3965, which was washed out before IL-4 stimulation, decreased M2 marker gene expression by IL-4 (ref. 33). Our explanation for their results is that intracellular cholesterol and its derivative oxysterols are considered to be decreased by the pretreatment of LXR agonist, leading to lower LXR activity during IL-4 stimulation, resulting in decreased expression of M2 markers. Thus, the report^33^ does not really conflict with our data. As for genetic ablation of LXR, direct *in vitro* testing on M2 polarization of LXR-deficient macrophages with IL-4 or IL-13 has not been reported, as far as we know. There is a report that high-fat-diet-fed C57BL/6 mice that had been transplanted the bone marrow of LXRα/β double KO mice were not affected with insulin resistance^34^, raising the possibility that LXR KO mice is not so largely affected by defective M2 polarization; we would like to point out that genetic ablation of LXR will result in loss of both repressor and enhancer roles of LXR, thus, LXR KO mice is not a perfect tool to investigate the roles of LXR. Our data showed that inactive LXR, namely antagonized-LXR or dominant-negative LXR, markedly blocked M2 polarization of wild-type BMDMs. The repressive effect of inactive LXR on M2 marker genes is compatible with the knowledge that ligand-free inactive LXR act as the repressor for gene expression^7^. We would like to propose LXR as a metabolic checkpoint in M0 macrophages to determine whether to be polarized into M2 macrophages or not: inactive LXR blocks M2 polarization, but active LXR allows M2 polarization.

From the perspective of metabolism, LXR is the master transcription factor that senses and controls cellular cholesterol amount^35^ by promoting the transcription of cholesterol-exporting molecules such as ABCA1. Thus, Lamtor1 not only integrates the amino-acid and IL-4 signals, but also couples the intracellular amino-acid sensing pathway and LXR, which is often referred to as a ‘cholesterol sensor'^36^. Because macrophages need cholesterol efflux to prevent accumulation of cholesterol that is derived from phagocytosed dead cells, it is not surprising that they express LXR at a high level. Indeed, relative to other tissues and cells, expression of LXRα and LXRβ was most prominent in macrophages (BioGPS^14^). As the roles of LXR in macrophages, suppression of inflammatory response^36^, differentiation of splenic marginal zone macrophages^35^, and promoting apoptotic cell clearance^37^ have been reported. These homoeostatic and immunoregulatory nature of LXR in macrophages seems compatible with our conclusion that active LXR is necessary for M2 polarization.

**Figure 8:**
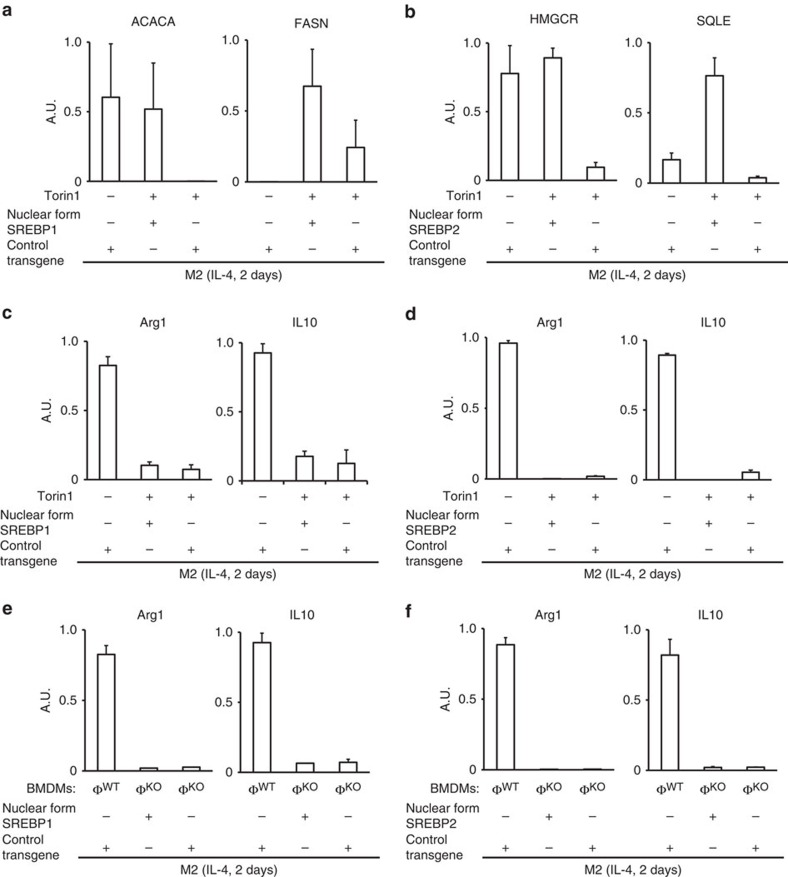
SREBPs are not involved in M2 polarization of macrophages. (**a**) Real-time PCR for SREBP1−target genes in IL-4−stimulated BMDMs. Nuclear form SREBP1 gene was introduced to wild-type BMDMs with retroviral vector. (**b**) Real-time PCR for SREBP2−target genes in IL-4−stimulated BMDMs. Nuclear form SREBP2 gene was introduced to wild-type BMDMs with retroviral vector. (**c**) Real-time PCR showed that forced expression of nuclear form SREBP1 did not rescue M2 polarization of mTOR-inhibited wild-type BMDMs. (**d**) Real-time PCR showed that forced expression of nuclear form SREBP2 did not rescue M2 polarization of mTOR-inhibited wild-type BMDMs. (**e**) Real-time PCR showed that forced expression of nuclear form SREBP1 did not rescue M2 polarization of Lamtor1-deficient BMDMs. (**f**) Real-time PCR showed that forced expression of nuclear form SREBP2 did not rescue M2 polarization of Lamtor1-deficient BMDMs. Φ^WT^ and Φ^KO^ are defined as in Fig. 1. The retroviral control transgene expressed GFP. More detailed information about SREBP1 and SREBP2 gene transduction is described in Method section. Error bars show s.d.

Complementally, we would like to mention SREBPs. Two previous reports showed that mTORC1 controls lipid metabolism via SREBPs^38^,^39^. One study using NIH3T3 cells showed that an mTOR inhibitor Torin1 blocked gene expression by SREBPs^39^, which controls lipid synthesis. We confirmed that expressions of SREBP-target genes were decreased after the treatment with Torin1, but rather enhanced following the loss of Lamtor1 (Supplementary Table 1). In Torin1-treated wild-type BMDMs, retroviral transfer of the nuclear form SREBP1 or SREBP2 rescued expression of SREBP-target genes (Fig. 8a,b), but did not rescue M2 signature genes (Fig. 8c,d). Forced expression of the nuclear form SREBP1 or SREBP2 also failed to rescue M2 polarization of Lamtor1-deficient BMDMs (Fig. 8e,f). These data indicated that gene expression by SREBPs is regulated by mTORC1, as reported^39^, but is not involved in M2 polarization of macrophages. Thus, our results expand the concept on the regulation of lipid metabolism by mTORC1, showing that not only gene expression by SREBPs, but also gene expression by LXR require active mTORC1 in macrophages.

**Figure 9:**
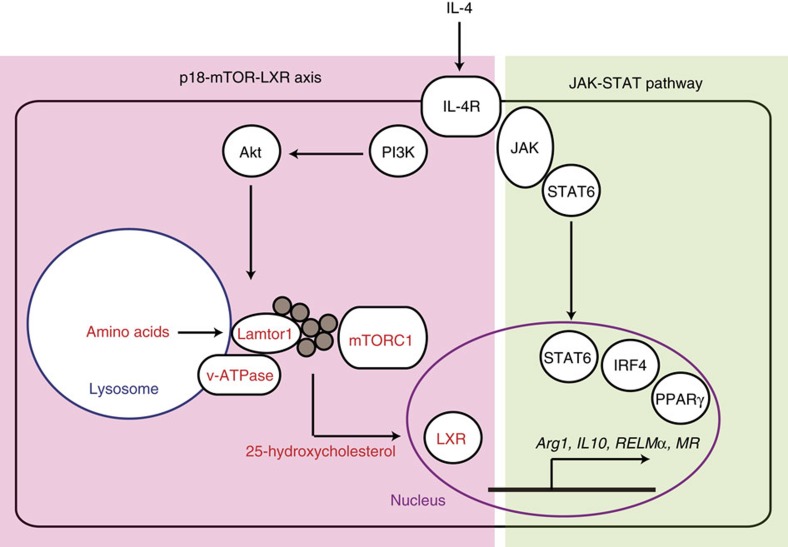
Scheme of the present study. In addition to previously identified JAK−STAT6 pathway, IRF4 and PPARγ, we demonstrated the essential role of a metabolic signaling axis consisting of the amino-acid sensing pathway (Lamtor1, v-ATPase and mTORC1) and downstream LXR in polarization of M2 macrophages.

In summary, we found that the lysosomal adaptor protein Lamtor1 is essential for the polarization of M2 macrophages both *in vitro* and *in vivo*. Lamtor1, v-ATPase and mTORC1 integrates the intracellular amino-acid sufficiency signal and the extrinsic IL-4 signal, leading to production of 25-hydroxycholesterol and subsequent activation of LXR, ultimately resulting in polarization of M2 macrophages (schematically depicted in Fig. 9).

## Methods

### Mice

Generation of *Lamtor1*^flox^ mice was described previously^40^. Genotyping for *Lamtor1*^flox^ and wild-type *Lamtor1* alleles was done with the reported primers (5′AAGGATTCGGAGTTAGAGACTAGGAC and 5′TGAGGATTCGAGTGGTGAGATACGA)^40^. *LysM-Cre* transgenic mouse^41^ was provided by Dr Shizuo Akira, and genotyping was performed with the primers: 5′CTTGCTGTGTGTTGTTCTGTGCTGAGG and 5′CGCATAACCAGTGAAACAGCATTGC. Myeloid-specific Lamtor1 conditional KO mice were generated by crossing the two strains, and then backcrossed 10 times to the C57BL/6J background. All myeloid-specific Lamtor1 conditional KO mice used in this study was *Lamtor1*^flox/flox^, *LysM-Cre* (heterozygote) mouse and littermate control mice were *Lamtor1*^flox/flox^ mice. Wild-type C57BL/6J mice were purchased from CLEA Japan. Mice were housed in specific pathogen−free conditions, and all experiment was performed according to the regulations of Osaka University. Eight to sixteen-week-old mice were used for experiments, except for the experiment shown in Supplementary Fig. 2. Both male and female mice were used; however, sex was always matched in each experiment.

### Macrophages

Bone marrow-derived macrophages (BMDMs) were differentiated as previously described^42^. In brief, bone marrow cells from femur and tibia were subjected to red blood cell lysis, and the surviving cells were cultured for 6 days in differentiation medium. The differentiation medium comprised of Dulbecco's minimum essential medium (Nacalai Tesque, Japan; 50% of total volume), L929 cell culture supernatant (30%), and heat-inactivated FCS (20%). Penicillin/streptomycin was also added. Developed BMDMs were polarized as M1 or M2 macrophages by 2 days of culture with the indicated stimulants: 20 ng ml^−1^ of recombinant mouse IFN-γ (485-MI; R&D Systems, MN, USA) and 100 ng ml^−1^ of LPS (L3024, Sigma-Aldrich, MO, USA) for M1 polarization; and 50 ng ml^−1^ of recombinant mouse IL-4 (404-ML, R&D Systems) for M2 polarization. In some experiments, BMDMs were polarized with stimulations above for 1 day or 8 h. To exclude unintended effect of L929 cell culture supernatant on BMDMs, we also differentiated BMDMs with recombinant M-CSF (216-MCC, R&D Systems), and confirmed the same results.

### Materials

Concanamycin A (C9705), Bafilomycin A1 (B1793), Wortmannin (W1628), LY294002 (L9908), Rapamycin (R8781), ion exchange chromatography−purified LPS (L3024) and 25-hydroxycholesterol (H1015) were purchased from Sigma-Aldrich; Torin1 (#4247) and GW3965 (#2474) from Tocris, UK; 5C-PPSS50 (#036-21321) from Wako, Japan; Akt inhibitor II (124008) from Merck Millipore, MA, USA.

### RNA and cDNA

Total RNA was purified using the RNeasy Mini Kit (Qiagen, Germany). cDNA was synthesized using the SuperScript III First-Strand Synthesis System (Invitrogen, MA, USA), which includes random hexamers as primers for reverse transcription. All procedures were performed according to the manufacturers' protocols.

### Quantitative PCR

We used commercial probes, master mix, and instruments supplied by Applied Biosystems, MA, USA: TaqMan probes for Lamtor1 (Mm01279964), Arg1 (Mm00475988), Resistin-like molecule alpha (Mm00445109), IL10 (Mm00439616), MR (Mm01329362), NOS2 (Mm00440502), IL6 (Mm00446190), IL12p40 (Mm01288989), TNF-α (Mm00443258), ABCA1 (Mm00442646), LPL (Mm00434764), HMGCR (Mm01282499), SQLE (Mm00436772), FASN (Mm00662319), ACACA (Mm01304257), LXRα (Mm00443451), LXRβ (Mm00437265), MerTK (Mm00434920), and GAPDH (#99999915); and TaqMan Gene Expression Master mix (#4369016). Real-time PCR was performed on a 7900HT Fast Real-Time PCR System, operated by the SDS 2.4 software. All gene expression level was normalized with the level of GAPDH in the same sample.

### Starvation for amino acids or glucose

Dulbecco's minimum essential medium (DMEM) (#08458-45, Nacalai Tesque) was used as the culture medium for BMDMs. For amino acid starvation, we used custom-made DMEM that lacks all amino acids (Cell Science & Technology Institute, Japan): the amount of each ingredient, except for amino acids, was matched with the control DMEM (Nacalai, above). For glucose starvation, commercially available glucose-free DMEM (042-32255, WAKO, Japan) was purchased. In starvation experiments, serum was not added to either control or starvation medium.

### Cell lysate preparation and western blot

For whole−cell lysates, lysis buffer A (50 mM Tris−HCl (pH 7.4), 150 mM NaCl, 1 mM EDTA, 5% glycerol, 2% n-octyl-β-D-glucopyranoside, and 1% Nonidet P-40 (NP-40)) was used as previously reported^13^. To prepare cytosolic and nuclear fractions, we modified the fractionation method that was distributed as a user resource by Abcam, UK. BMDMs were suspended in hypotonic buffer B (20 mM Tris-HCl (pH 7.4), 10 mM NaCl, 3 mM MgCl_2_) and incubated 10 min on ice; next, a final concentration of 0.5% NP-40 was added and the sample was vortexed for 10 s. After 1-min incubation on ice, the sample was again vortexed for 10 s, and then centrifuged 10 min at 3,000 r.p.m. in a microcentrifuge (5424R, Eppendorf, Germany). The supernatant was used as the cytosolic fraction, and the pellet containing nuclei was lysed with RIPA buffer (25 mM Tris–HCl (pH 7.6), 150 mM NaCl, 1% NP-40, 1% sodium deoxycholate, 0.1% sodium dodecyl sulfate). Successful fractionation was confirmed and shown in Supplementary Fig. 8a. For western blots, reduced protein samples for SDS-PAGE were prepared by boiling 1:1 mixtures of cell lysates and 2 × Laemmli sample buffer containing 2-mercaptoethanol. Antibodies for western blots were purchased from indicated suppliers: S6K (#2708), p-S6K (T389, #9234), Akt (#4691), p-Akt (Ser473, #4060), p-Akt (Thr308, #9275), Lamtor1(#8975), Lamtor2 (#8145), Lamtor3 (#8168), Lamtor4 (#12284), Lamtor5 (#14633), α-tubulin (2144), and PPARγ (#2443) from Cell Signaling Technology, MA, USA; IRF4 (sc-6059), arginase-1 (sc-20150), LXRα/β (sc-13068), Lamin B (sc-6216) and β-actin (clone AC-15) from Santa Cruz Biotechnology; resistin-like molecule alpha (ab39628), STAT6 (ab44718), p-STAT6 (Y641, ab54461) and ABCA1 (ab18180) from Abcam; anti-FLAG (A8592) from Sigma-Aldrich; and GAPDH from ThermoFisher Scientific, MA, USA. Horse-radish peroxidase−conjugated secondary antibodies were purchased from GE Healthcare, UK and DAKO (Agilent Technologies, CA, USA). Working concentrations of antibodies are listed in Supplementary Table 2. Confirmation of specificity of antibodies is shown in Supplementary Fig. 9. Signals were detected using ImageQuant LAS 500 (GE Healthcare). In Fig. 5b,f, density of each bands was quantified with ImageJ, and the densities of p-S6K bands standardized by that of S6K bands were shown in the figures.

### Flow cytometry

For screening of immune system development, 6−8-week-old mice were analysed. Thymocytes were stained with CD4 (clone RM4-5) and CD8 (clone 53-6.7) antibodies. Splenocytes were stained with CD3 (clone 145-2C11), CD4, CD8, B220 (clone RA3-6B2), CD11b (clone M1/70) and Ly6G (clone RB6-8C5) antibodies. Bone marrow cells were stained with CD3, B220, CD11b and Ly6G antibodies. For the confirmation of development of BMDMs, antibodies for CD11b, F4/80 (clone BM8) and MHC class II (I-A/I-E; clone M5/114.15.2) were used. Cells were analysed on a FACS Canto II (BD Biosciences). Appropriate gates for FSC and SSC were set to analyse each population. Working concentrations of antibodies are listed in Supplementary Table 2.

### Gene transfer to macrophages

The coding sequence for mouse Lamtor1, dominant-negative mouse LXRα (ref. 24) (1–435 amino acid residues), and dominant-negative mouse LXRβ (ref. 24) (1–437 amino acid residues) were cloned into pMX-IRES-GFP (Cell Biolabs, CA, USA). The coding sequence for murine nuclear form SREBP1 or SREBP2 (1–480 and 1–473 amino acid residues, respectively) was fused with the sequence for FLAG-tag and cloned into pRetroX-TetOne (Clontech, CA, USA). All plasmid vectors were checked with Sanger sequencing to exclude mutations. To prepare retroviral vectors, Platinum-E cells (Cell Biolabs) were used as packaging cells. BMDMs were cultured as described above, and culture supernatant of Platinum-E cells containing retroviral vector was added on day 3. For selection of BMDMs harbouring the doxycycline-inducible SREBP1 or SREBP2 genes, empty pMX-IRES-GFP retroviral vector was simultaneously infected as a fluorescent marker. On day 6, gene-transferred macrophages were sorted as GFP^high^ cells, which were clearly distinct from the GFP−negative population, using a FACS Aria III (BD Biosciences, CA, USA). If necessary, 200 ng ml^−1^ of doxycycline was added to the culture media during M2 polarization after sorting. See Supplementary Fig. 8e for the confirmation of the protein expression from pRetroX-TetOne vector.

### Challenge with LPS *in vivo*

For Fig. 2c, two groups of mice, consisting of four littermate controls or four conditional KO mice, were intravenously administered with LPS (L3024, Sigma). After the indicated times, one mouse from each group was sacrificed to collect whole blood. In survival experiment, mice were observed without any sampling. For measurement of serum HMGB1, non-lethal dose of LPS for Lamtor1 conditional KO mice was administered intravenously.

### Challenge with Zymosan A

A dose of 200 μg of Zymosan A (Z4250, Sigma-Aldrich) in PBS(−) was intravenously administered to mice. Sera were obtained 6 h after injection.

### Enzyme-linked immunosorvent assay

Serum cytokine levels were measured with the Quantikine ELISA kit (R&D Systems), and cytokines in culture supernatant were measured with DuoSet ELISA development systems (R&D Systems). HMGB1 was measured with an ELISA kit purchased from SHINO-TEST, Japan. All procedure was performed according to the manufacturers' protocols.

### *In vivo* induction of M2 macrophages

Brewer's thioglycollate medium (#225710, BD Biosciences) was diluted 4% (w/v) in distilled water, and then autoclaved. One millilitre of the diluted medium per mouse was injected into the intraperitoneal cavity. After 6 days, peritoneal cells were collected. Intracellular staining was performed with the Cytofix/Cytoperm Cell Permeabilization/Fixation Solution (BD Biosciences). Antibodies used for defining M2 macrophages were resistin-like molecule alpha (ab39628), mannose receptor (clone MR5D3) and CD11b (clone M1/70). Working concentrations of antibodies are listed in Supplementary Table 2. To define MR^+^ and RELMα^+^ cells, positive gates for these markers were set to contain <1% of the negative control samples stained with fluorescence-labelled isotype control IgG. Cells were analysed on a FACS Canto II (BD Biosciences).

### Cell size measurement

Cell sizes were estimated by flow cytometry. Forward scatter intensities measured on a FACS Canto II were considered to reflect cell size.

### Gene expression microarray

Total RNA of the macrophages from WT and KO mice was extracted RNeasy Mini kit (Qiagen) according to the manufacturer's instructions. RNAs were quantified using a Nanodrop ND-1000 spectrophotometer (Thermo Scientific) RNA integrity was evaluated using an RNA 6000 LabChip and Bioanalyzer (Agilent Technologies). Each RNA with RNA integrity numbers greater than 9.7 was used for microarray experiments. Two hundred nanogram of total RNA was reverse transcribed into double strand cDNAs by AffinityScript multiple temperature reverse transcriptase and amplified for 2 h at 40 °C. Resulting cDNAs were subsequently used for *in vitro* transcription by T7-polymerase and labelled with cyanine-3-labelled cytosine triphosphate (Perkin-Elmer) for 2 h at 40 °C using a Low Input Quick-Amp Labelling Kit ver. 6.5 (Agilent Technologies) according to the manufacturer's protocol. After labelling, rates of dye incorporation and quantification were measured with a Nanodrop ND-1000 spectrophotometer (Thermo Scientific) and were then fragmented for 30 min at 60 °C in the dark. The labelled 600 ng of each cRNA sample was then hybridized on Agilent 8 × 60 K whole mouse genome arrays (Agilent Design # 028005) at 65 °C for 17 h with rotation in the dark. Hybridization was performed using a Gene Expression Hybridization Kit (Agilent Technologies) following the manufacturer's instructions. After washing in GE washing buffer, slides were scanned with an Agilent Microarray Scanner G2505C. Feature extraction software (Version 10.5.1.1) employing defaults for all parameters was used to convert images into gene expression data.

### Microarray design

Expression profiling was generated using 8 × 60 K whole mouse genome oligo microarray G4852A (Agilent Technologies). Each microarray uses 55,681 probes to interrogate 39,430 Entrez gene RNAs and 16,251 lincRNAs. Four microarray analyses as 1-colour microarray-based gene expression analysis were performed with wild-type M1-polarized BMDMs (M1), wild-type M2-polarized BMDMs (M2) (polarization was done as described above), IL-4−stimulated Lamtor1-deficient BMDMs (KO) and Torin1-treated and IL-4−stimulated wild-type BMDMs (Torin1). Each gene expression profiling was compared between M1/M2, KO/M2 and Torin1/M2.

### Microarray data analysis

Raw data were imported into the Subio Platform and Subio Basic Plug-in (v1.11; Subio Inc., Aichi, Japan) for quality control and calculating the between-sample *fold change*. For normalization, the threshold raw signal was set to 1.0 and the global normalization algorithm (percentile shift) was used with a percentile target of 75. This normalized data was used for identifying differentially expressed genes, in which M1, KO or Torin1 were compared with M2 as control. Raw data have been accepted in Gene Expression Omnibus (GEO), a public repository for microarray data, aimed at storing Minimum Information About Microarray Experiments (MIAME).

### Mass spectrometry for oxysterols

The LC/MS analysis was conducted using a *Nexera* UHPLC system connected to a triple quadrupole mass spectrometer LCMS-8060 (Shimadzu, Kyoto, Japan). The sample solutions extracted by methanol were kept at 5 °C and 5 μl was taken for the LC/MS analysis. A reversed-phase core shell column (CAPCELL CORE C27, 2.1 × 75 mm, 2.7 μm, Shiseido, Japan) was used for chromato-graphic separation maintained at 40 °C. Five mM ammonium formate in water and methanol were used for mobile phase A and B, respectively, with a flow rate of 0.3 ml min^−1^. Concentration of mobile phase B was programmed as 20% (0 min)—20% (1 min)—40% (2 min)—85% (10 min)—100% (10.1 min)—100% (15 min)—20% (15.1 min)—20% (20 min) including curved binary gradient (–5) from 2 min (40% B) to 10 min (85% B). The mass spectrometric parameters were set as follows: nitrogen gas was used for nebulizer- and drying gases were set at 3 and 10 l min^−1^, respectively. Dry air was used for heating gas with 10 l/min. Argon gas (purity, >99.9995%) was used for collision-induced dissociation (CID). Interface, heat block and desolvation line temperatures were kept at 300 °C, 400 °C and 250 °C, respectively. We selected dehydrated molecular ion, (M+H−H_2_O)^+^, as the precursor ions for following multiple reaction monitoring (MRM) transitions; 385.3>159.1, 385.3>161.1 and 392.3>147.1 for 25-hydroxycholesterol, 27-hydroxycholesterol, and 24(R/S)-hydroxycholesterol-d7, respectively. Internal standard method was performed for quantification of 25-hydroxycholesterol and 27-hydroxycholesterol in the calibration range from 10 to 1,000 ng ml^−1^. 25-hydroxycholesterol and the other isomers such as 24(S)-hydroxycholesterol detected at *m/z* 385.3>159.1 were differentiated by the chromatographic separation as shown in Supplementary Fig. 10.

### Preparation of apoptotic thymocytes

Similar to previous reports, thymuses were obtained from 6-week-old C57BL/6J mice, then grinded with sterilized slide glasses in Hanks's balanced salt solution. Thymocytes were then collected by centrifugation, subsequently diluted and cultured in RPMI medium supplemented with 10% FBS, 5% penicillin-streptomycin, and final 50 μM of 2-mercaptoethanol. Apoptosis was induced by overnight culture with final 200 nM of dexamethasone (D4902, Sigma-Aldrich) in the medium.

### Luciferase assay

Luciferase expression vector under the control of the LXR-response element in the promoter (#336841, Qiagen) was transfected into HEK293 cells using Lipofectamine 2000 (Invitrogen). After 48 h, the transfected cells were treated with 250 nM of Torin1 for indicated times. Luciferase activity was measured with the Dual-Glo Luciferase assay system (E2920, Promega, WI, USA) and an ARVO MX 1420 (Perkin-Elmer, MA, USA). Samples were measured in quintuplicate.

### Statistics

The *P* values of Student's *t*-test were calculated with Microsoft Excel 2010 or EZR (Jichi Medical University, Saitama, Japan)^43^. Log-rank test was performed with EZR, a graphical user interface for R (The R Foundation for Statistical Computing, Austria).

### Data availability

Gene expression microarray data have been deposited in GEO under the accession code GSE51665. The authors declare that all data supporting the findings of this study are available within the article and its Supplementary Information files or are available from the corresponding author upon request.

## Additional information

**How to cite this article:** Kimura, T. *et al*. Polarization of M2 macrophages requires Lamtor1 that integrates cytokine and amino-acid signals. *Nat. Commun.* **7**, 13130 doi: 10.1038/ncomms13130 (2016).

## Supplementary Material

Supplementary Information Supplementary Figures 1-10 and Supplementary Tables 1-2.

Supplementary Data 1 Lists of genes in microarray; It is a multi-tabbed excel file.

## References

[b1] Abbas A., Lichtman A. & Pillai S. in Cellular and Molecular Immunology 8th edn Ch.4, p73Elsevier (2014).

[b2] Sica A. & Mantovani A. Macrophage plasticity and polarization: *in vivo* veritas. J. Clin. Invest. 122, 787–795 (2012). 22378047 10.1172/JCI59643 PMC3287223

[b3] Murray P. J. . Macrophage activation and polarization: nomenclature and experimental guidelines. Immunity 41, 14–20 (2014). 25035950 10.1016/j.immuni.2014.06.008 PMC4123412

[b4] Martinez F. O., Helming L. & Gordon S. Alternative activation of macrophages: an immunologic functional perspective. Annu. Rev. Immunol. 27, 451–483 (2009). 19105661 10.1146/annurev.immunol.021908.132532

[b5] Nagata S. Apoptosis and autoimmune diseases. Ann. N. Y. Acad. Sci. 1209, 10–16 (2010). 20958310 10.1111/j.1749-6632.2010.05749.x

[b6] Chantranupong L., Wolfson R. L. & Sabatini D. M. Nutrient-sensing mechanisms across evolution. Cell 161, 67–83 (2015). 25815986 10.1016/j.cell.2015.02.041 PMC4384161

[b7] Calkin A. C. & Tontonoz P. Transcriptional integration of metabolism by the nuclear sterol-activated receptors LXR and FXR. Nat. Rev. Mol. Cell. Biol. 13, 213–224 (2012). 22414897 10.1038/nrm3312 PMC3597092

[b8] Maderna P. & Godson C. Phagocytosis of apoptotic cells and the resolution of inflammation. Biochim. Biophys. Acta (BBA)—Mol. Basis Dis. 1639, 141–151 (2003). 10.1016/j.bbadis.2003.09.004 14636945

[b9] Voll R., Herrmann M., Roth E., Stach C. & Kalden J. Immunosuppressive effects of apoptotic cells. Nature 390, 350 (1997). 9389474 10.1038/37022

[b10] Sancak Y. . Ragulator-Rag complex targets mTORC1 to the lysosomal surface and is necessary for its activation by amino acids. Cell 141, 290–303 (2010). 20381137 10.1016/j.cell.2010.02.024 PMC3024592

[b11] Menon S. . Spatial control of the TSC complex integrates insulin and nutrient regulation of mTORC1 at the lysosome. Cell 156, 771–785 (2014). 24529379 10.1016/j.cell.2013.11.049 PMC4030681

[b12] Bar-Peled L., Schweitzer, Lawrence D., Zoncu R. & Sabatini D. M. Ragulator is a GEF for the Rag GTPases that signal amino acid levels to mTORC1. Cell 150, 1196–1208 (2012). 22980980 10.1016/j.cell.2012.07.032 PMC3517996

[b13] Nada S. . The novel lipid raft adaptor p18 controls endosome dynamics by anchoring the MEK-ERK pathway to late endosomes. EMBO J. 28, 477–489 (2009). 19177150 10.1038/emboj.2008.308 PMC2657578

[b14] Wu C. . BioGPS: an extensible and customizable portal for querying and organizing gene annotation resources. Genome Biol. 10, R130 (2009). 19919682 10.1186/gb-2009-10-11-r130 PMC3091323

[b15] Wang J. & Kubes P. A reservoir of mature cavity macrophages that can rapidly invade visceral organs to affect tissue repair. Cell 165, 668–678 (2016). 27062926 10.1016/j.cell.2016.03.009

[b16] Lotze M. T. & Tracey K. J. High-mobility group box 1 protein (HMGB1): nuclear weapon in the immune arsenal. Nat. Rev. Immunol. 5, 331–342 (2005). 15803152 10.1038/nri1594

[b17] Odegaard J. I. . Macrophage-specific PPARgamma controls alternative activation and improves insulin resistance. Nature 447, 1116–1120 (2007). 17515919 10.1038/nature05894 PMC2587297

[b18] Zoncu R. . mTORC1 senses lysosomal amino acids through an inside-out mechanism that requires the vacuolar H(+)-ATPase. Science 334, 678–683 (2011). 22053050 10.1126/science.1207056 PMC3211112

[b19] Thoreen C. C. . An ATP-competitive mammalian target of rapamycin inhibitor reveals rapamycin-resistant functions of mTORC1. J. Biol. Chem. 284, 8023–8032 (2009). 19150980 10.1074/jbc.M900301200 PMC2658096

[b20] Byles V. . The TSC-mTOR pathway regulates macrophage polarization. Nat. Commun. 4, 2834 (2013). 24280772 10.1038/ncomms3834 PMC3876736

[b21] Ruckerl D. . Induction of IL-4Ralpha-dependent microRNAs identifies PI3K/Akt signaling as essential for IL-4-driven murine macrophage proliferation *in vivo*. Blood 120, 2307–2316 (2012). 22855601 10.1182/blood-2012-02-408252 PMC3501641

[b22] Sancak Y. . The rag GTPases bind raptor and mediate amino acid signaling to mTORC1. Science 320, 1496–1501 (2008). 18497260 10.1126/science.1157535 PMC2475333

[b23] Noguchi-Yachide T. . Structural development of liver X Receptor (LXR) antagonists derived from thalidomide-related glucosidase inhibitors. Chem. Pharm. Bull. 55, 1750–1754 (2007). 18057753 10.1248/cpb.55.1750

[b24] Venkateswaran A. . Control of cellular cholesterol efflux by the nuclear oxysterol receptor LXR alpha. Proc. Natl Acad. Sci. USA 97, 12097–12102 (2000). 11035776 10.1073/pnas.200367697 PMC17300

[b25] Ganeshan K. & Chawla A. Metabolic regulation of immune responses. Annu. Rev. Immunol. 32, 609–634 (2014). 24655299 10.1146/annurev-immunol-032713-120236 PMC5800786

[b26] Weichhart T., Hengstschlager M. & Linke M. Regulation of innate immune cell function by mTOR. Nat. Rev. Immunol. 15, 599–614 (2015). 26403194 10.1038/nri3901 PMC6095456

[b27] Covarrubias A. J. . Akt-mTORC1 signaling regulates Acly to integrate metabolic input to control of macrophage activation. eLIFE 5, e11612 (2016). 26894960 10.7554/eLife.11612 PMC4769166

[b28] Weichhart T. . The TSC-mTOR signaling pathway regulates the innate inflammatory response. Immunity 29, 565–577 (2008). 18848473 10.1016/j.immuni.2008.08.012

[b29] Jenkins S. J. . Local macrophage proliferation, rather than recruitment from the blood, is a signature of TH2 inflammation. Science 332, 1284–1288 (2011). 21566158 10.1126/science.1204351 PMC3128495

[b30] Dibble C. C. & Manning B. D. Signal integration by mTORC1 coordinates nutrient input with biosynthetic output. Nat. Cell. Biol. 15, 555–564 (2013). 23728461 10.1038/ncb2763 PMC3743096

[b31] Pourcet B. . LXRalpha regulates macrophage arginase 1 through PU.1 and interferon regulatory factor 8. Circ. Res. 109, 492–501 (2011). 21757649 10.1161/CIRCRESAHA.111.241810 PMC3180895

[b32] Kiss E. . Suppression of chronic damage in renal allografts by Liver X receptor (LXR) activation relevant contribution of macrophage LXRalpha. Am. J. Pathol. 179, 92–103 (2011). 21703396 10.1016/j.ajpath.2011.03.019 PMC3123851

[b33] Chao L. C. . Bone marrow NR4A expression is not a dominant factor in the development of atherosclerosis or macrophage polarization in mice. J. Lipid. Res. 54, 806–815 (2013). 23288947 10.1194/jlr.M034157 PMC3617954

[b34] Marathe C. . Preserved glucose tolerance in high-fat-fed C57BL/6 mice transplanted with PPARgamma^−/−^, PPARdelta^−/−^, PPARgammadelta^−/−^, or LXRalphabeta^−/−^ bone marrow. J. Lipid. Res. 50, 214–224 (2009). 18772483 10.1194/jlr.M800189-JLR200 PMC2636915

[b35] Gonzalez N. A. . The nuclear receptor LXRalpha controls the functional specialization of splenic macrophages. Nat. Immunol. 14, 831–839 (2013). 23770640 10.1038/ni.2622 PMC3720686

[b36] Joseph S. B., Castrillo A., Laffitte B. A., Mangelsdorf D. J. & Tontonoz P. Reciprocal regulation of inflammation and lipid metabolism by liver X receptors. Nat. Med. 9, 213–219 (2003). 12524534 10.1038/nm820

[b37] A-Gonzalez N. . Apoptotic cells promote their own clearance and immune tolerance through activation of the nuclear receptor LXR. Immunity 31, 245–258 (2009). 19646905 10.1016/j.immuni.2009.06.018 PMC2791787

[b38] Zeng H. . mTORC1 couples immune signals and metabolic programming to establish T(reg)-cell function. Nature 499, 485–490 (2013). 23812589 10.1038/nature12297 PMC3759242

[b39] Peterson T. R. . mTOR complex 1 regulates lipin 1 localization to control the SREBP pathway. Cell 146, 408–420 (2011). 21816276 10.1016/j.cell.2011.06.034 PMC3336367

[b40] Soma-Nagae T. . The lysosomal signaling anchor p18/LAMTOR1 controls epidermal development by regulating lysosome-mediated catabolic processes. J. Cell. Sci. 126, 3575–3584 (2013). 23781028 10.1242/jcs.121913

[b41] Takeda K. . Enhanced Th1 activity and development of chronic enterocolitis in mice devoid of Stat3 in macrophages and neutrophils. Immunity 10, 39–49 (1999). 10023769 10.1016/s1074-7613(00)80005-9

[b42] Kobayashi K. . IRAK-M is a negative regulator of toll-like receptor signaling. Cell 110, 191–202 (2002). 12150927 10.1016/s0092-8674(02)00827-9

[b43] Kanda Y. Investigation of the freely available easy-to-use software ‘EZR' for medical statistics. Bone Marrow Transplant. 48, 452–458 (2013). 23208313 10.1038/bmt.2012.244 PMC3590441

