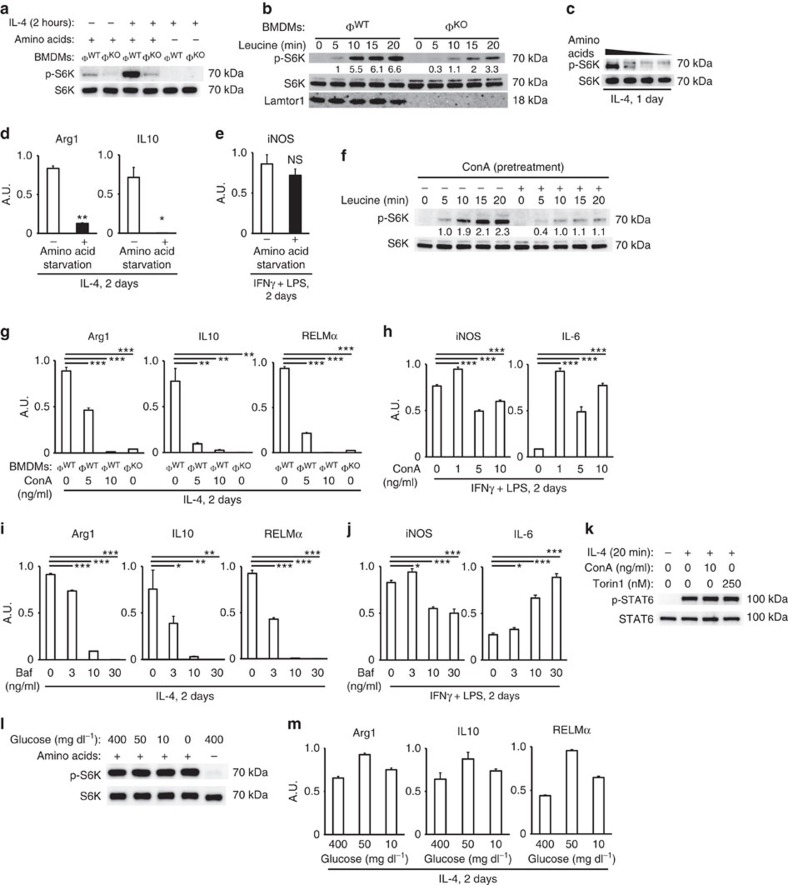# Erratum: Polarization of M2 macrophages requires Lamtor1 that integrates cytokine and amino-acid signals

**DOI:** 10.1038/ncomms14711

**Published:** 2017-02-27

**Authors:** Tetsuya Kimura, Shigeyuki Nada, Noriko Takegahara, Tatsusada Okuno, Satoshi Nojima, Sujin Kang, Daisuke Ito, Keiko Morimoto, Takashi Hosokawa, Yoshitomo Hayama, Yuichi Mitsui, Natsuki Sakurai, Hana Sarashina-Kida, Masayuki Nishide, Yohei Maeda, Hyota Takamatsu, Daisuke Okuzaki, Masaki Yamada, Masato Okada, Atsushi Kumanogoh

Nature Communications 7 Article number: 13130; DOI: 10.1038/ncomms13130 (2016); Published 10 12 2016; Updated 02 27 2017

In this Article, there are errors in the labelling of the *x* axis in Fig. 5g,h that were introduced during the production process. The second label on the *x* axis of each graph in Fig. 5g should have been ‘_Φ_WT' instead of ‘_Φ_KO'. In Fig. 5h, the labels ‘0', '5', '10' and ‘0' should have been ‘0‘, ‘1', '5' and ‘10', respectively. In addition, lane labels indicating the duration of leucine stimulation are missing from the Western blot in Fig. 5b, and should have read ‘0', ‘5', ‘10', ‘15', ‘20,' ‘0', ‘5', ‘10', ‘15' and ‘20', from left to right. The correct version of Fig. 5 appears below as Fig. 1.

## Figures and Tables

**Figure 1:**