# Supplementary material for: Polarization of M2 macrophages requires Lamtor1 that integrates cytokine and amino-acid signals
Source: Nat Commun. 2016 Oct 12;7:13130. doi: 10.1038/ncomms13130 (PMC5064021; doi:10.1038/ncomms13130)
Supplement: Supplementary Information — Supplementary Figures 1-10 and Supplementary Tables 1-2. [file ncomms13130-s1.pdf]

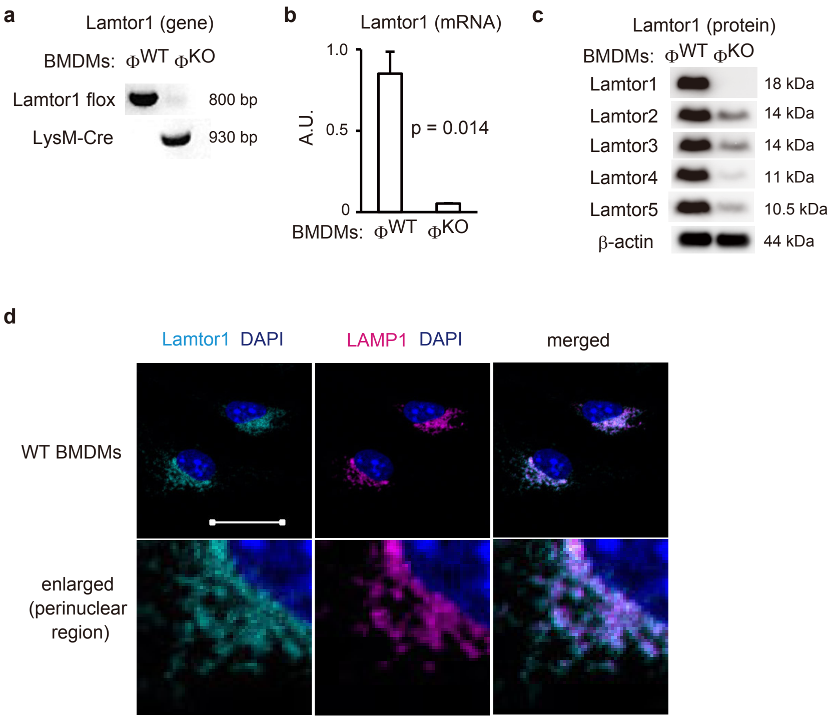

Supplementary Figure 1 | Generation of myeloid-specific Lamtor1 conditional knockout mice, and subcellular localization of Lamtor1 in macrophages.

(a-c) Confirmation of Lamtor1 knockout in macrophages. BMDMs that were generated with L929 cell culture supernatant were used as sample. All macrophages used in this figure were M0 macrophages (i.e. before M1 or M2 polarization). (a) PCR for Lamtor1^{flox} allele and LysM-Cre transgene in genomic DNA of macrophages confirmed successful deletion of Lamtor1 gene in macrophages. (b) Real-time PCR for Lamtor1 mRNA also confirmed the knockout of Lamtor1. (c) Western blots confirmed knockout of Lamtor1, and revealed the accompanied reduction of Lamtor2-5 proteins in Lamtor1-deficient BMDMs. (d) Confocal microscopy for Lamtor1 and LAMP1 (lysosome/late endosome marker) in wild-type BMDMs showed the localization of Lamtor1 at those organelles. Two macrophages are shown in upper panels; nuclei were stained with DAPI. The scale bar incicates 20 micrometers. A magnified perinuclear region is shown in lower panels.

ΦWT and ΦKO: BMDMs generated from the bone marrow of Lamtor1$^{flox/flox}$, or Lamtor1$^{flox/flox}$ LysM-Cre mice, respectively. The representative results of two or three independent experiments are shown for each panel. Error bars show standard deviation

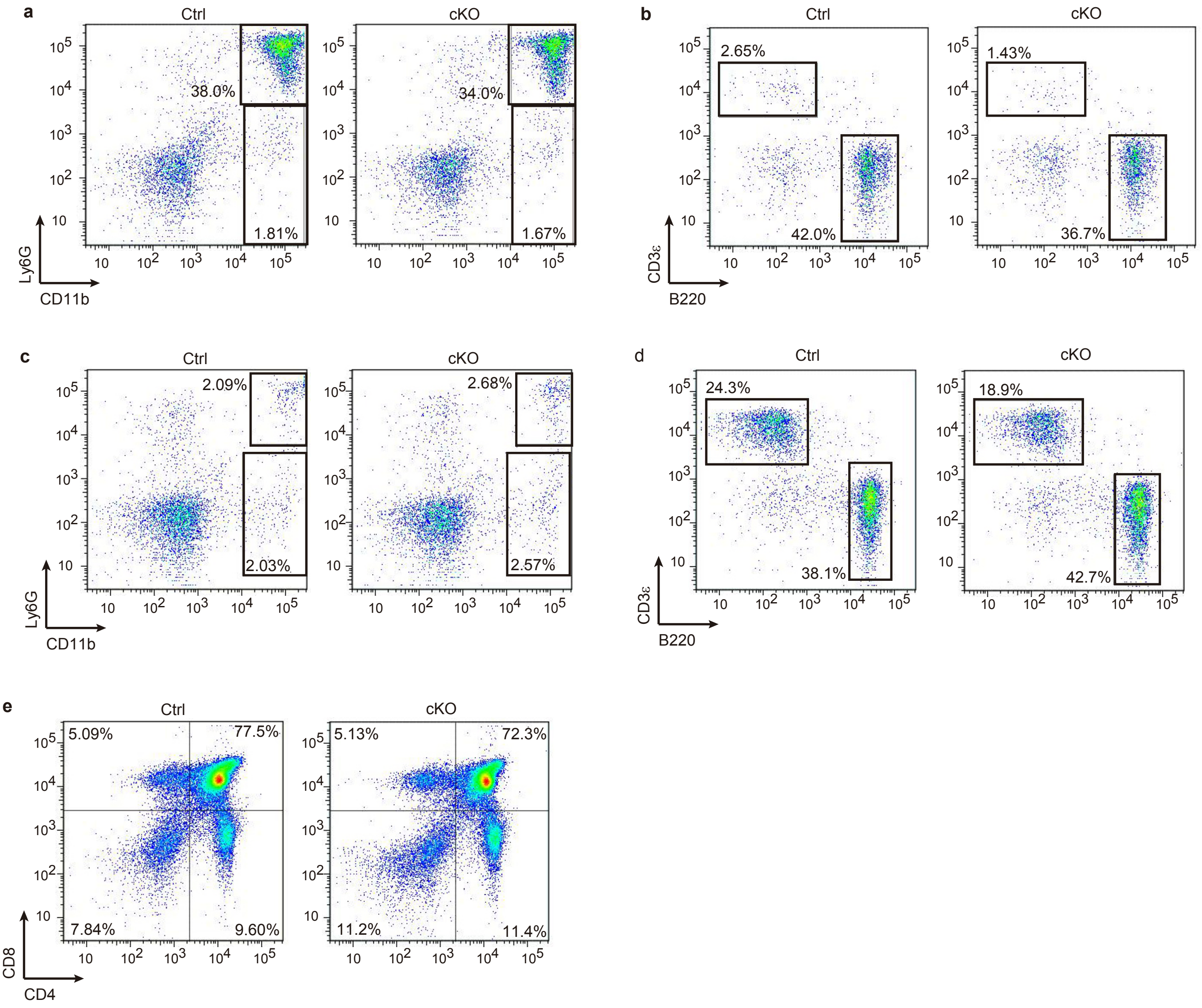

Supplementary Figure 2 | Comparable development of immune systems in myeloid-specific Lamtor1 conditional knockout mice and their littermate controls.

Flow cytometry for (a) myeloid cells (neutrophils: CD11b^{+} Ly6G^{high}; monocytes/macrophages CD11b^{+} Ly6G^{dull}) and (b) lymphocytes (T cells: CD3ε^{+}; B cells: B220^{+}) in the bone marrow; (c) myeloid cells (neutrophils: CD11b^{+} Ly6G^{high}; monocytes/macrophages CD11b^{+} Ly6G^{dull}) and (d) lymphocytes (T cells: CD3ε^{+}; B cells: B220^{+}) in the spleen; and (e) T cell development in the thymus. Six to eight-week-old mice were used.

Ctrl: *Lamtor1*$^{flox/flox}$; cKO: *Lamtor1*$^{flox/flox}$ *LysM-Cre* mice. The representative results of three independent experiments are shown for each panel.

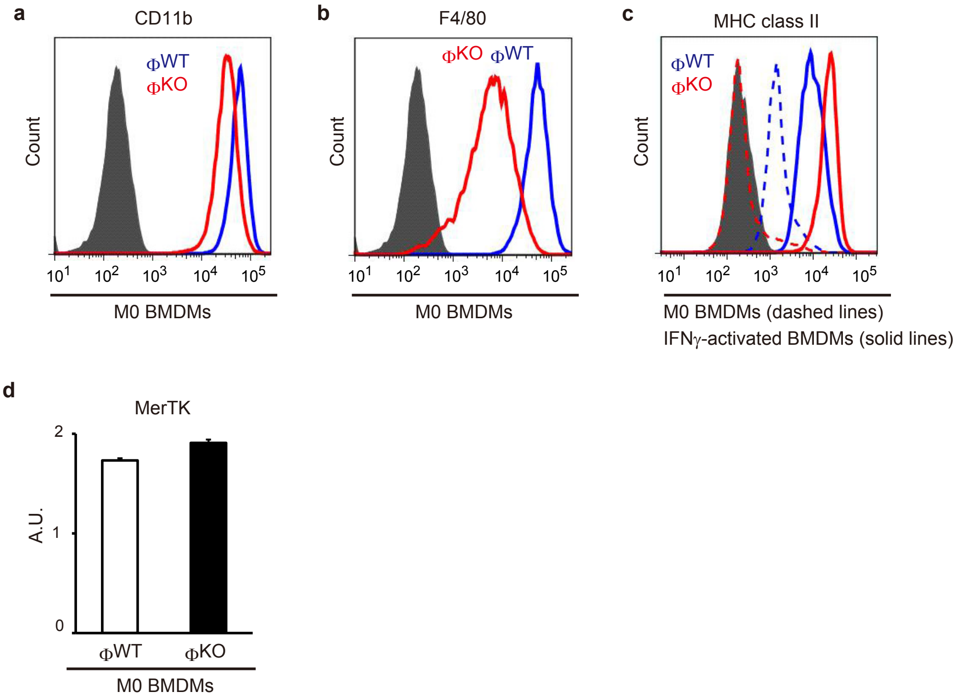

Supplementary Figure 3 | Macrophage markers were expressed in Lamtor1-deficient macrophages. Flow cytometry for (a) a myeloid marker CD11b, (b) a macrophage marker F4/80, and (c) an antigen presenting molecule MHC class II expressed on the surface of BMDMs. Expression of MHC class II (I-A/I-E) was examined in both M0 BMDMs and in BMDMs that had been activated by overnight stimulation with 20 ng/ml of IFNγ. (d) Real-time PCR for another macrophage marker MerTK.

ΦWT and ΦKO are defined as in Figure 1.

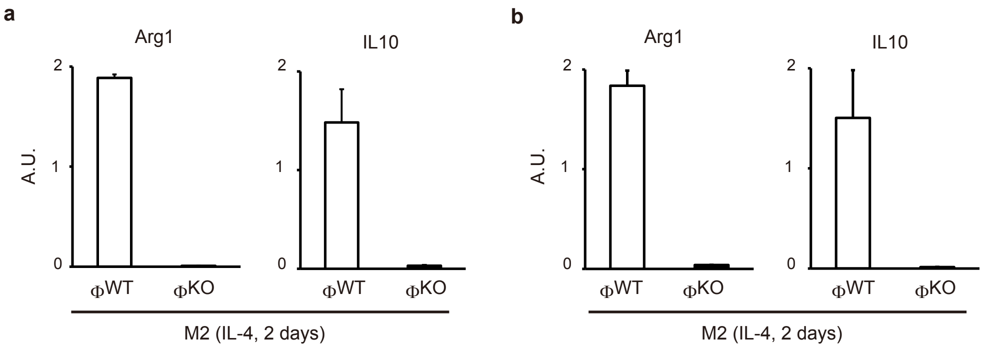

Supplementary Figure 4 | Defective M2 polarization of p18-deficient BMDMs.

BMDMs differentiated with L929 cell culture supernatant (a) and BMDMs differentiated with recombinant M-CSF (b) were polarized to M2 macrophages by IL-4 stimulation. The defective M2 polarization of p18-deficient BMDMs was consistently observed regardless of the differentiation method.

ΦWT and ΦKO are defined as in Figure 1.

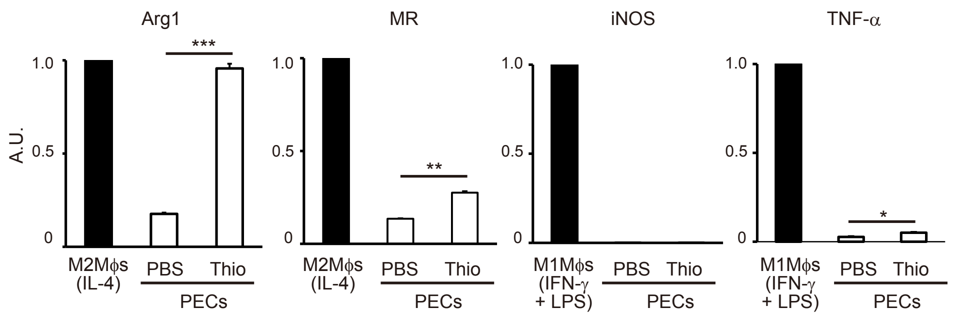

Supplementary Figure 5 | M2 markers were expressed in thioglycollate-induced peritonitis. Real-time PCR for M1 and M2 markers confirmed that M2 polarization is the major activation mode at 6 days after the intraperitoneal injection of thioglycollate medium (related to Fig. 2a). Positive controls are shown as black bars (in vitro-polarized M1 or M2 wild-type BMDMs), and mRNAs obtained from whole peritoneal white blood cells (PECs) are shown in white bars. Intraperitoneal administration of thioglycollate medium (Thio) to C57BL/6J mouse increased M2 markers in PECs, compared to administration of PBS.

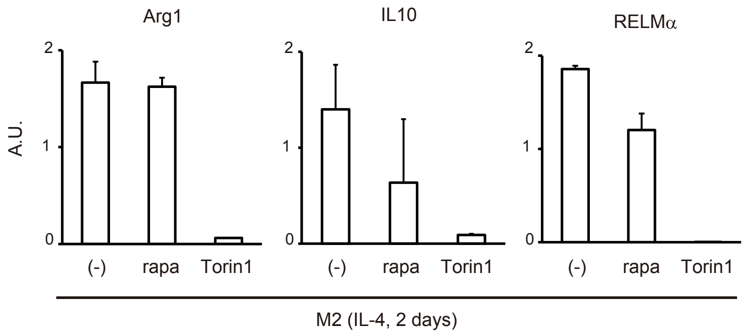

Supplementary Figure 6 | Torin1 blocked expression of M2 markers, but rapamycin did not. Real-time PCR for expression of M2 marker genes. Torin1 (250 nM) decreased expression of M2 markers in IL-4–stimulated wild-type BMDMs. Rapamycin (rapa; 50 ng/ml) weakly suppressed expression of some M2 markers in IL-4–stimulated wild-type BMDMs. Inhibitors existed during the two days of IL-4 stimulation.

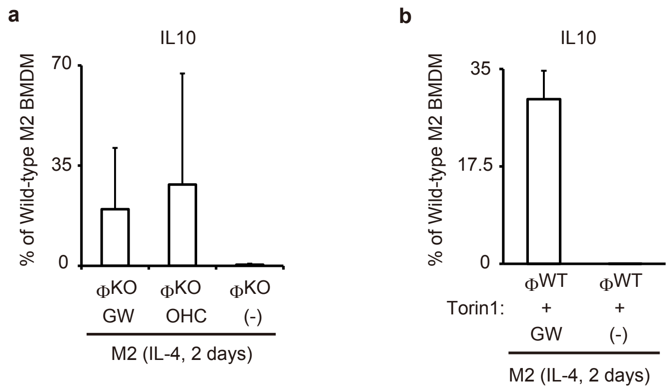

Supplementary Figure 7 | LXR agonists rescued expression of an M2 marker IL10 in p18-deficient BMDMs and Torin1-treated wild-type BMMs.

Real-time PCR for expression level of IL10. (a) LXR agonists GW3965(GW) and 25-hydroxycholesterol(OHC) rescued expression of IL10 in IL-4 stimulated Lamtor1-deficient BMDMs. (b) An LXR agonist GW3965 rescued expression of IL10 in IL-4–stimulated and Torin1–treated wild-type BMDMs.

Expression levels were shown as percentages, compared to the level in IL-4-stimulated wild-type BMDMs.ΦWT and ΦKO are defined as in Figure 1.

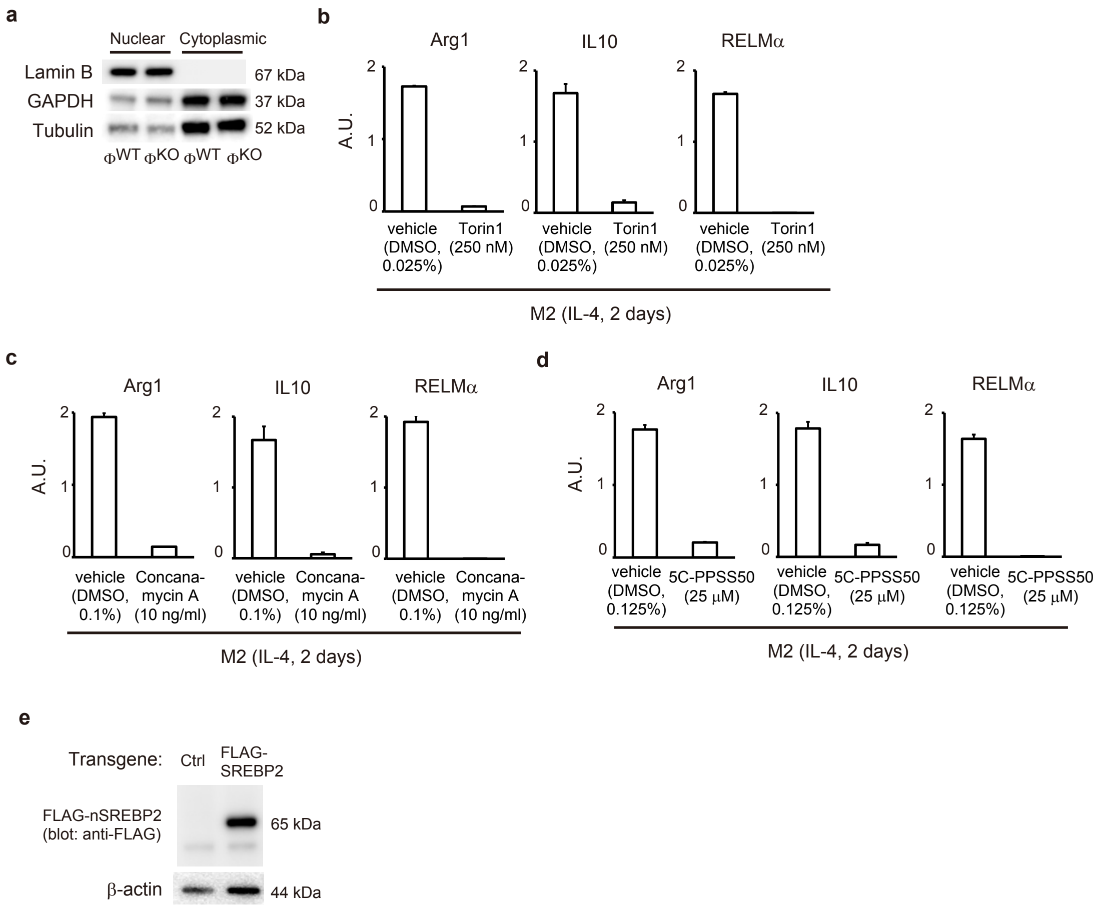

Supplementary Figure 8 | Confirmation for experimental procedures.

(a) Western blots for confirmation of the fractionation method for nuclear and cytosolic fractions (related to Fig. 3a.). See Method section for detailed procedure of the fractionation. (b) Real-time PCR for M2 marker genes expressed in wild-type BMDMs treated with Torin1 or matched dose of the vehicle DMSO. M2 polarization was abolished by Torin1, not by non-specific effect of DMSO. (related to Fig. 3f). (c) Real-time PCR for M2 marker genes expressed in wild-type BMDMs treated with concanamycin A or matched dose of the vehicle DMSO. M2 polarization was abolished by concanamycin A, not by non-specific effect of DMSO. (related to Fig. 5g). (d) Real-time PCR for M2 marker genes expressed in wild-type BMDMs treated with 5C-PPSS50 or matched dose of the vehicle DMSO. M2 polarization was abolished by 5C-PPSS50, not by non-specific effect of DMSO. (related to Fig. 7a). (e) Western blot confirmed the forced expression of nuclear form SREBP protein by retroviral gene transfer (related to Figure 8). The band size was slightly larger than 60 kDa.

* $p<0.05$, ** $p<0.01$, *** $p<0.001$. Error bars show standard deviation.

Supplementary Figure 9 |

Confirmation of specificity of antibodies.

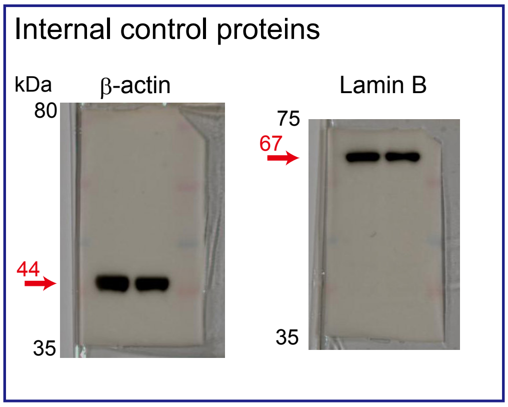

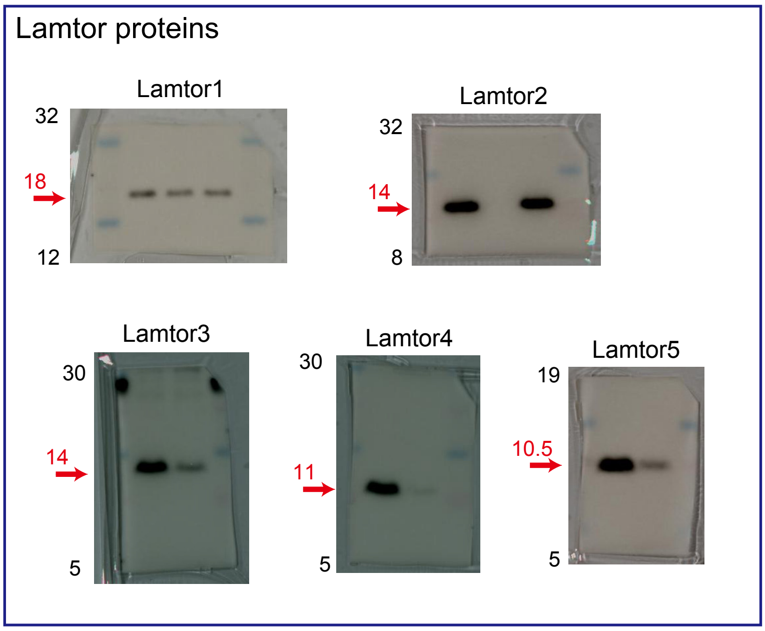

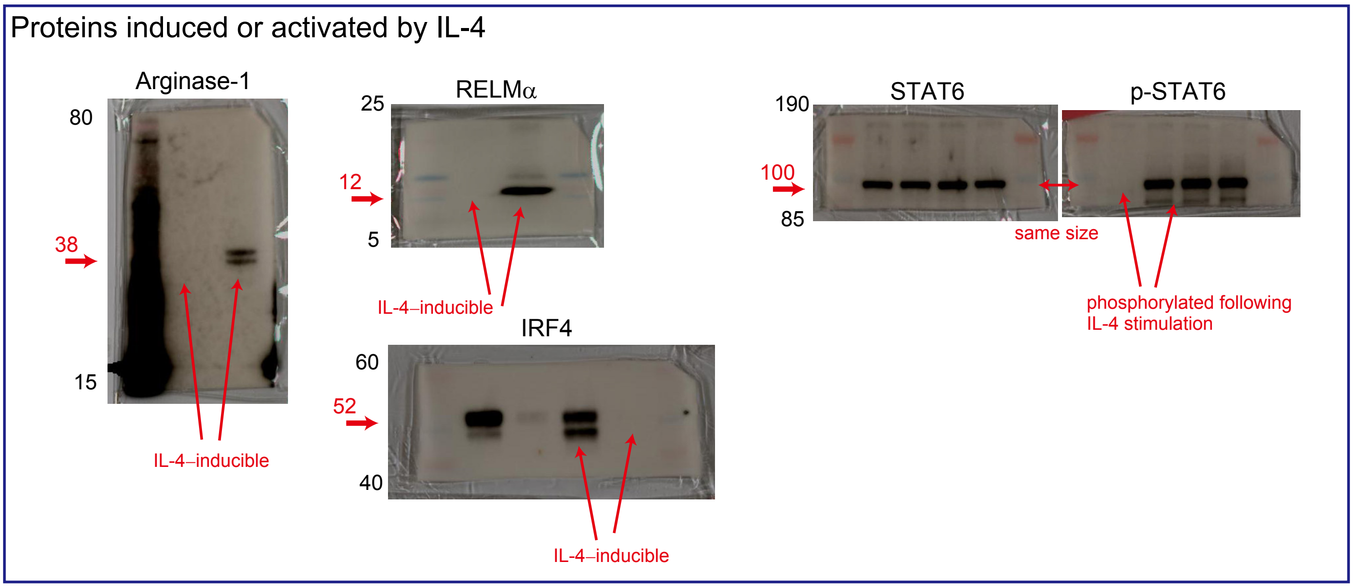

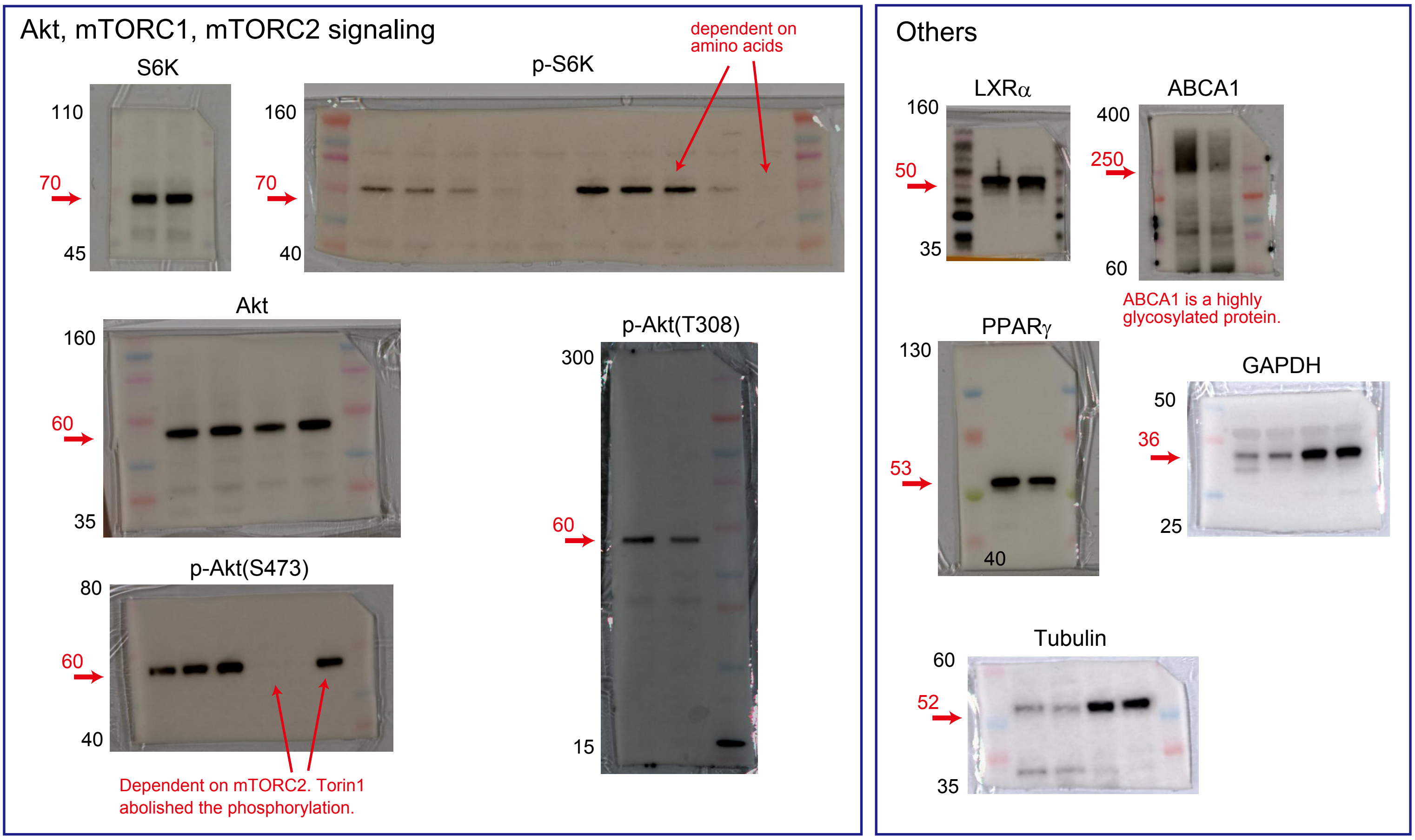

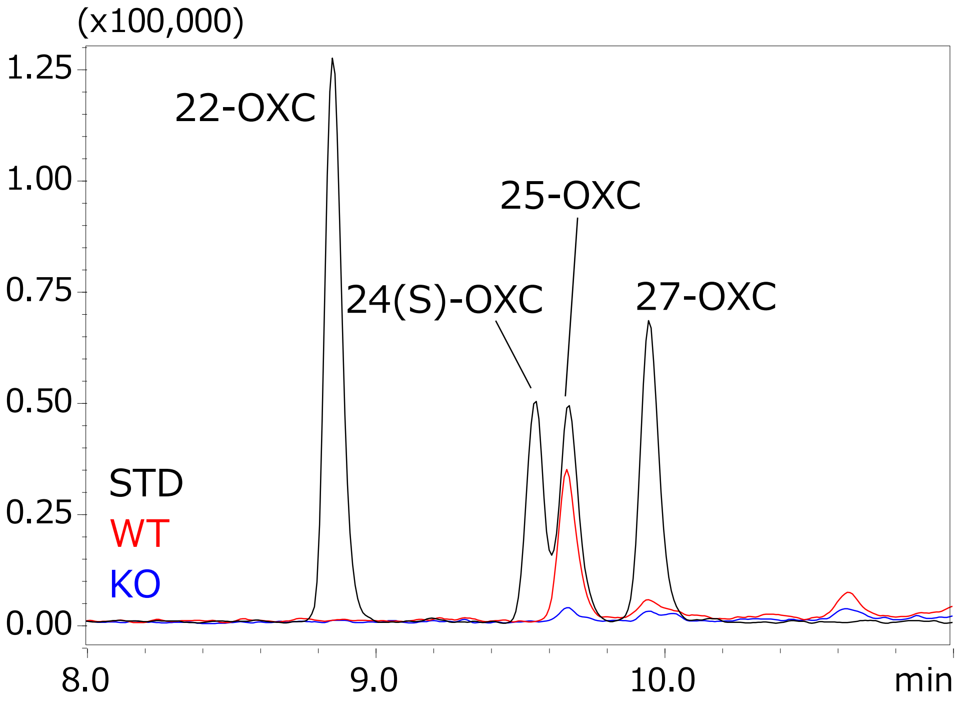

Supplementary Figure 10 | MRM chromatograms monitored by the transition of *m/z* 385.3 > 159.1 for hydroxycholesterol standards mixture (black), wild type (red) and knock out (blue) were overlaid. Retention times of 25-hydroxychoresterol (25-OXC) were 9.56 min and 9.67 min, respectively.

Supplementary Table 1 | Expression of SREBP-target genes in IL-4–stimulated BMDMs.

Gene	Wild-type BMDM	Lamtor1–deficient BMDM	Torin1–treated WT BMDM
FASN	53	147	27
ACACA	4,223	7,781	975
SCD1	124	2,284	24
HMGCR	511	685	137
FDPS	11,701	23,980	2,124
SQLE	4,882	8,018	421

Values in gene expression microarray

Supplementary Table 2

Concentrations of antibodies

Antigen	Supplier	Catalog# or Clone	Application	Dilution
S6K	Cell Signaling Technology	2708	WB	3,000
p-S6K (T389)	Cell Signaling Technology	9234	WB	2,000
Akt	Cell Signaling Technology	4691	WB	3,000
p-Akt (S473)	Cell Signaling Technology	4060	WB	2,000
p-Akt (T308)	Cell Signaling Technology	9275	WB	2,000
Lamtor1/p18	Cell Signaling Technology	8975	WB	2,000
Lamtor2	Cell Signaling Technology	8145	WB	2,000
Lamtor3	Cell Signaling Technology	8168	WB	2,000
Lamtor4	Cell Signaling Technology	12284	WB	2,000
Lamtor5	Cell Signaling Technology	14633	WB	2,000
PPAR gamma	Cell Signaling Technology	2443	WB	2,000
IRF4	Santa Cruz Biotechnology	sc-6059	WB	2,000
Arginase-1	Santa Cruz Biotechnology	sc-20150	WB	2,000
LXR	Santa Cruz Biotechnology	sc-13068	WB	2,000
Lamin B	Santa Cruz Biotechnology	sc-6216	WB	3,000
beta-actin	Santa Cruz Biotechnology	clone AC-15	WB	10,000
Resistin-like molecule alpha	Abcam	ab39628	WB	500
STAT6	Abcam	ab44718	WB	3,000
p-STAT6 (Y641)	Abcam	ab54461	WB	2,000
ABCA1	Abcam	ab18180	WB	2,000
GAPDH	ThermoFisher	AM4300	WB	3,000
alpha-tubulin	Cell Signaling Technology	2144	WB	2,000
FLAG (HRP-conjugated Ab)	Sigma	A8592	WB	10,000
Goat Immunoglobulin	DAKO	P0449	WB (2'Ab; HRP)	100,000
Mouse IgG	GE	NA931V	WB (2'Ab; HRP)	100,000
Rabbit IgG	GE	NA934V	WB (2'Ab; HRP)	50,000
CD3	Biolegend	clone 145-2C11	Flow cytometry	50
CD4	Biolegend	clone RM4-5	Flow cytometry	50
CD8	Biolegend	clone 53-6.7	Flow cytometry	50
CD11b	Biolegend	clone M1/70	Flow cytometry	50
B220	eBioscience	clone RA3-6B2	Flow cytometry	50
Ly6G	eBioscience	clone RB6-8C5	Flow cytometry	50
Mannose receptor	BD	clone MR5D3	Flow cytometry	50
Resistin-like molecule alpha	Abcam	ab39628	Flow cytometry	50
F4/80	Biolegend	clone BM8	Flow cytometry	50
MHC class II (I-A/I-E)	eBioscience	clone M5/114.15.2	Flow cytometry	50
